# Assessment of pollution status using Water Quality Index (WQI) and hydrochemical ındicators in the Gemlik Gulf, Marmara Sea, Türkiye: a spatial and temporal perspective

**DOI:** 10.1007/s11356-025-36560-8

**Published:** 2025-06-02

**Authors:** Efsun Dindar

**Affiliations:** Department of Environmental Engineering, Faculty of Engineering, Bursa Uludag University, 16059 Bursa, Turkey

**Keywords:** Eutrophication, Gemlik Gulf, Seawater, Water quality

## Abstract

Coastal regions, including the Gemlik Gulf in Turkey, face increasing threats from environmental pollution, driven by industrial activities, urban expansion, and wastewater discharges. Despite its ecological and economic significance, limited studies have comprehensively assessed the spatiotemporal variations in seawater quality in this region. This study aims to fill this gap by evaluating the key pollution sources affecting seawater quality in the Gemlik Gulf, particularly following the 2021 Marmara Sea mucilage crisis. The research investigates the distribution of various seawater quality parameters in the Gemlik Gulf (GG), including pH, temperature, density, fluorescence, NH_4_-N, NOx (NO_2_ + NO_3_), total N (TN), total P (TP), salinity, chlorophyll-a (Chl-a), and dissolved oxygen (DO). Samples were collected from 31 stations between January 2022 and November 2022, covering the Spring, Summer, Autumn, and Winter seasons. During the investigation, the temperature, salinity, and dissolved oxygen levels of the seawater varied from 10.3–27.3 °C, 23–38.54 psu, and 2.63–10.57 mg L^−1^, respectively. The amounts of nitrate + nitrite-N (0.01–3.356 µM), TN (125.72–271.1 µg L^−1^), and TP (4.62–28.88 µg L^−1^) were measured. Chl-a levels ranged from 0.259 to 6.44 µg L^−1^. Surface water quality can be investigated and classified based on contamination levels using the Water Quality Index (WQI) and trophic status. The WQI classed seawater samples as medium water quality. However, the WQI was specifically calculated for surface waters discharging into the sea. The results indicated that Engürücük River (WQI = 78) and Susurluk River (WQI = 77) were classified as “very poor,” while Nilüfer River (WQI = 50) and Karsak River (WQI = 57) were categorized as “poor.” This situation indicates that the rivers carry significant pollution to the Sea of Marmara. According to the Surface Water Quality Management Regulation, the Gemlik Gulf was classified as hypertrophic-eutrophic in terms of TP and NOx, between January and April varied by location, but was predominantly determined as oligotrophic-mesotrophic. Based on Chl a and trophic index, it was determined that the gulf generally had low water quality-high mesotrophic and poor water quality-eutrophic conditions during winter.

**Supplementary Information:**

The online version contains supplementary material available at 10.1007/s11356-025-36560-8.

## Introduction

Numerous factors affect seawater quality, affecting its composition and appropriateness for diverse uses. The salinity, temperature, dissolved oxygen (DO), pH, nutrients, microorganisms, and other elements that make up seawater quality are important for human activities like fishing, aquaculture, and tourism as well as for the wellbeing of marine ecosystems. These are some of the important parameters used to assess seawater quality. In order to detect any changes and take appropriate action, management and conservation initiatives in coastal and marine regions usually require routine monitoring of these parameters (Koronides et al. 2024; Yousefi et al. 2019). Between the Mediterranean and the Black Sea is the semi-enclosed Marmara Sea. Surface and deep currents from the Black and Mediterranean Seas bring pollutants into the Marmara Sea through the water. Low salinity (18–29‰) Black Sea-derived water makes up the surface layer. High salinity (∼ 38.5‰) Mediterranean Sea water makes up the remaining water layer (below 20–25 m) (Shiganova et al. 1995; Lee et al. 2002). Numerous industrial complexes, municipal wastes, agricultural chemicals, oil pollution, and airborne particles have all contributed to the extremely high levels of pollution that have been observed in the Marmara Sea (Tuğrul and Polat 1995; Yümün and Ekici 2023).

Terrestrial organic matter arising as a result of anthropogenic activity (Cetecioğlu et al. 2009), which has caused the pollution in the Marmara Sea can precipitate from the surface to the lower sea layer and stimulate microbial activity and organic matter degradation (Evcen et al. 2024; Ritta et al. 2010). A previous study (Balkis et al. 2012; Balci and Balkis 2017) mentioned that nutrients might come from the same sources into the water column because of the strong positive correlation between nitrogen, PO_4_-P, and SiO_4_-Si in the Gulfs of Bandırma and Erdek (Marmara Sea) and also noted to increase in nutrient concentration at the depths of 30 m as a result of the bacterial decomposition of the organic substances aggregating at the bottom.

The Sea of Marmara is surrounded by five metropolitan municipalities and two provincial municipalities, most of which treat their wastewater physically to separate coarse and fine particulates. Then, using a liquid waste disposal process called deep-sea discharge, these effluents enter the Sea of Marmara (Burak et al. 2021). This method attempts to take advantage of the sea’s inherent dilution and cleansing processes. Wastewater is piped and diffused to the seabed at various distances from the shoreline. Decarbonization plants and advanced biological treatment facilities do exist; however, they are in the minority (Maryam and Büyükgüngör, 2019). Many studies have shown that this “deep discharge” practice has caused an excess of the nutrient load that exceeds the capacity of the Marmara’s marine ecosystem (Okuş et al. 2002; Çardak et al. 2015; Taş et al. 2020).

Additionally, the broader water quality issues present in the Marmara Sea are further intensified within the Gulf. In this context, identifying the factors affecting the water quality in the Gemlik Gulf, monitoring pollution levels, and planning necessary interventions to preserve the ecological balance of the region are of utmost urgency. However, existing studies often focus on local pollution sources, lacking a holistic evaluation of water quality and its implications for ecosystem health.

Meriç et al. (2005) describe the Gemlik Gulf as a basin with a depth of 110 m that is divided from the Marmara Sea by a ridge that is 50 m deep. The geographic structure of the Gulf influences both biotic and abiotic environmental forces. Moreover, organic matter precipitates from the Marmara Sea’s surface to its lower layer, aided by microbial activity, enriching the nutrient-deficient waters that originate in the Mediterranean with inorganic nutrients (Tuğrul et al. 2002; Akçay 2022). This emphasizes the need to implement environmentally friendly practices, such as treating wastewater, protecting coastlines, and giving ecosystem health, water quality, ecosystem restoration, and biodiversity top priority. These practices are motivated by the critical roles that coastal marine biodiversity plays in regulating the global climate as a carbon sink, as well as in supplying food and sustaining a variety of commercial activities (Eichbaum et al. 1996; Daoji and Daler 2004; Kumar et al. 2023).

Efforts to monitor and manage seawater quality in the Marmara Sea have primarily focused on the use of various water quality indices (WQIs) and the implementation of regulations such as the European Water Framework Directive (WFD 2000/60/EC Directive). Using both biotic and abiotic factors, the European Water Framework Directive has established categorization standards for water quality (WFD 2020). Variations in biodiversity can be used to track the substantial effects of pollution on aquatic ecosystems (Ogidi and Akpan 2022; Cardinale 2011).

The Water Quality Index (WQI) is the most efficient technique to understand, manage, and assess water quality data (Yousefi et al. 2019; Radfard et al. 2019). In order to provide a thorough evaluation of overall water quality levels, this practical approach uses mathematical algorithms to combine large datasets on water quality into a single measure (Radfard et al. 2018). Additionally, a number of quality indices for coastal waters have been established (Zingone et al. 2021; Taş et al. 2020). These indices rely on physico-chemical factors to determine the trophic status. The utilisation of water quality indicators enables the evaluation of water quality in the Gulf of Gemlik, consequently supporting the detection of any hazards to human health and ecological stability.

The novelty of this research lies in its comprehensive assessment of the Gemlik Gulf’s water quality, particularly in the context of the 2021 Marmara Sea mucilage crisis, which severely impacted marine life, fisheries, and the regional economy. This study aims to evaluate the spatial and seasonal variations in seawater quality by employing a multi-metric approach, integrating the trophic status, Water Quality Index (WQI), and ecological quality.

Seawater samples were collected from multiple stations across different seasons in 2022, allowing for a systematic evaluation of pollution sources, nutrient dynamics, and ecosystem responses. The results are expected to enhance scientific understanding of post-mucilage water quality and provide critical insights for the development of evidence-based management strategies aimed at preserving marine biodiversity and promoting the sustainable use of coastal resources.

## Methods

### Study area

**Fig. 1:**
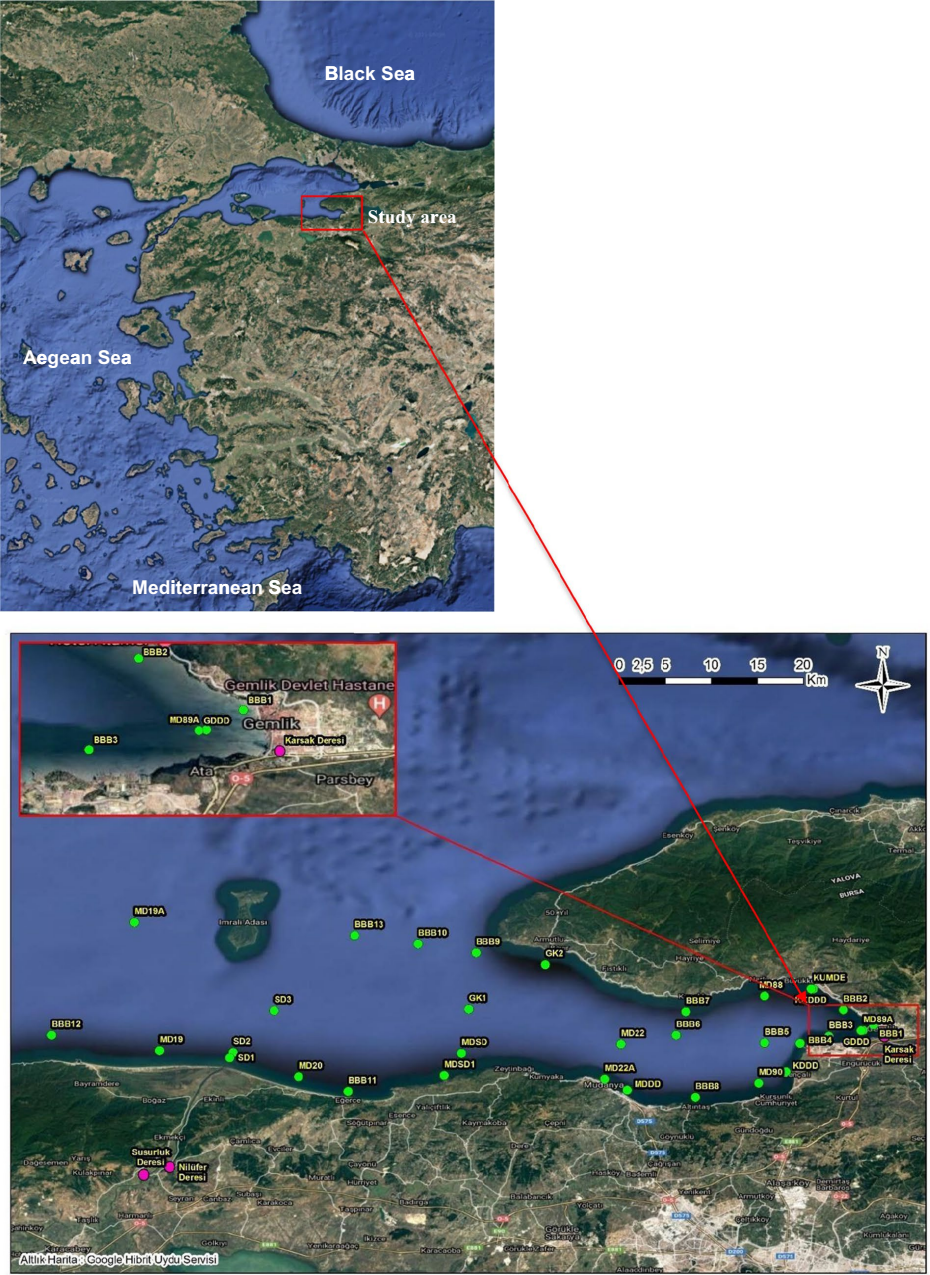
Study area and monitoring stations

The Gulf of Gemlik, which collects pollution from Lake Iznik as well as industrial and domestic garbage from adjacent towns, and Izmit Gulf, which receives waste from Turkey’s most important industrial district, are both critical coastline and near-shore areas in the Marmara Sea. The Gemlik Gulf is a basin that is roughly 35 km long and 15 km broad, and it is situated in the southeast corner of the Sea of Marmara’s southern shelf. The gulf’s deepest point is roughly 110 m (Fig. 1). More than 40 big and moderate industrial plants are concentrated along the Gulf’s eastern and southeast coastlines. Increasing vertical mixing during the winter months alters anoxic conditions with DO concentrations about 1.3 mg L^−1^, even when summertime DO values drop as low as 0.1–0.9 mg L^−1^ (Yüksek et al. 2004).

Due to the influx of Black Sea waters and the vertical mixing with nutrient-rich deep waters from the Mediterranean in the Marmara Sea, nutrient concentrations in surface waters rise between late autumn and mid-spring (Yılmaz 2002). The deep waters of the Mediterranean, deficient in nutrients and saline, travel through the Dardanelles and reach the southern shelf of the Marmara Sea (Tuğrul et al. 2002). Eventually, they cross a ridge and enter the deeper levels of the Gemlik Gulf.

The Gemlik Gulf is a southeastern part of the Marmara Sea that contains two different water masses: a deeper layer that comes from the Mediterranean and is sourced from the Black Sea, and an upper layer that is 10–15 m thick and has a salinity of 22–26 psu. The pycnocline, a sharp boundary with a thickness of roughly 10–20 m, separates these layers (Ünlüata et al. 1990). The gulf, which is 35 km long and 15 km wide, creates a semi-enclosed basin that is 110 m deep at its deepest point. A fault-controlled depositional area is located along its central trough, which is migrating northwest (Yaltırak and Alpar 2002; Kuşçu et al. 2009). This study was carried out seasonally in the Gemlik Gulf between January 2022 and December 2022 (Fig. 1).

### Sampling and preparation

The sampling, measurement, and analyses are determined according to national and international standards, as well as the “Regulation on Sampling and Analysis Methods for Water Pollution Control” and the guidelines of UNEP/FAO/IAEA/IOC/MAP/WHO (Table S1).

Water samples representing surface water were taken from a depth of 0.5 m, and for bottom water, samples were taken from the deepest point possible in the water column (3 m above the total depth of the station). Additionally, in stations where stratification (halocline) is apparent, samples were taken from the middle depth of the halocline region to represent the intermediate water layer. In stations where no stratification is observed (shallow stations from 20 to 40 m), only surface and bottom samples were taken, while sampling at three depths was carried out in relatively deep stations (deeper than 40 m).

Water quality parameters were determined using standard methods (APHA/AWWA/WEF 2017) At all stations, water column temperature, salinity, density/sigma-t, fluorescence, pH, and dissolved oxygen profiling were measured with multi parameter instruments on site (SeaBird brand SBE-25 Plus CTD) Secchi disk (SD) depths were measured with a 30 cm diameter white disk.

Chlorophyll-a (Chl-a) was analyzed spectrophotometrically according to SM 10200 H, NH_4_-N, and NO_3_-N determination was performed with the flow injection method by the SM 4500-NH₃ H, SM 4500-NO I, respectively. The total nitrogen (TN) content of the samples was analyzed with the autoanalyzer after persulfate digestion, as described by APHA, AWWA, and WEF (2017). Total phosphorus (TP) samples were digested with potassium persulfate to a reactive form in an autoclave and analyzed in the autoanalyzer according to the methods of SM 4500-P J.

### Methods

#### Calculation of Water Quality Index

The Water Quality Index (WQI) is a metric designed to represent the collective impact of various water quality parameters (Sahu and Sikdar 2008).

Seven parameters were chosen: pH, dissolved oxygen, nitrate, nitrite, ammonia, phosphate, and temperature. According to Sánchez et al. (2007), these are the primary characteristics required to determine the rank of water quality using the WQI calculator.

WQI = k ∑^n^_i=1_ CiWi

∑^n^_i=1_ W_i_

Sanchez et al. (2007), Varol (2020), Ustaoglu and Aydın (2020), Aydin et al. (2021), and Das Kangabam et al. (2017) all refer to the Water Quality Index (WQI) as a mathematical tool that combines various water characterization data into a single numerical representation of overall water quality, which is presented in the Supplementary Section (Table S2, Pesce and Wunderlin 2000; Kannel et al. 2007; and Koçer and Sevgili 2014).

In this equation, k is a subjective constant representing the visual judgment of water quality, Ci is the normalized value allocated to each measured parameter (Ustaoglu and Aydın, 2020), and Wi is the relative weight assigned to each parameter (WHO 2017). Water quality assessments are classified into five categories based on WQI values. Accordingly, WQI < 25 is excellent; 26 ≤ WQI < 50 is good; 51 WQI < 75 is poor; 76 ≤ WQI < 100 is very poor; WQI ≥ 100 is Fair (Ustaoglu et al. 2021).

### Data analysis

The Statistica software 12.0 was used in this study. Pearson correlation analysis, one-way ANOVA, cluster analyses were performed. Hierarchical cluster analysis was used to discover comparable groupings of cases or variables, and the similarity coefficient was calculated using Pearson's correlation matrix (Yang et al. 2020) (Table 1).

**Table 1:**
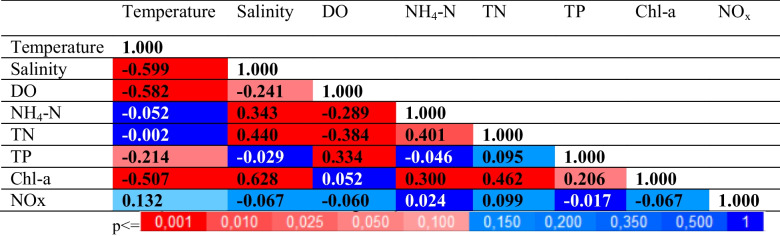
Pearson correlation matrix of water quality parameters for surface water

In order to determine whether the water quality parameters measured across different seasons and stations exhibited statistically significant spatial variation, one-way ANOVA was applied. When significant effects were indicated by ANOVA, post hoc analysis was performed using Tukey’s HSD multiple comparison test.

## Result and discussion

### Water quality assessment

#### Winter season

**Fig. 2:**
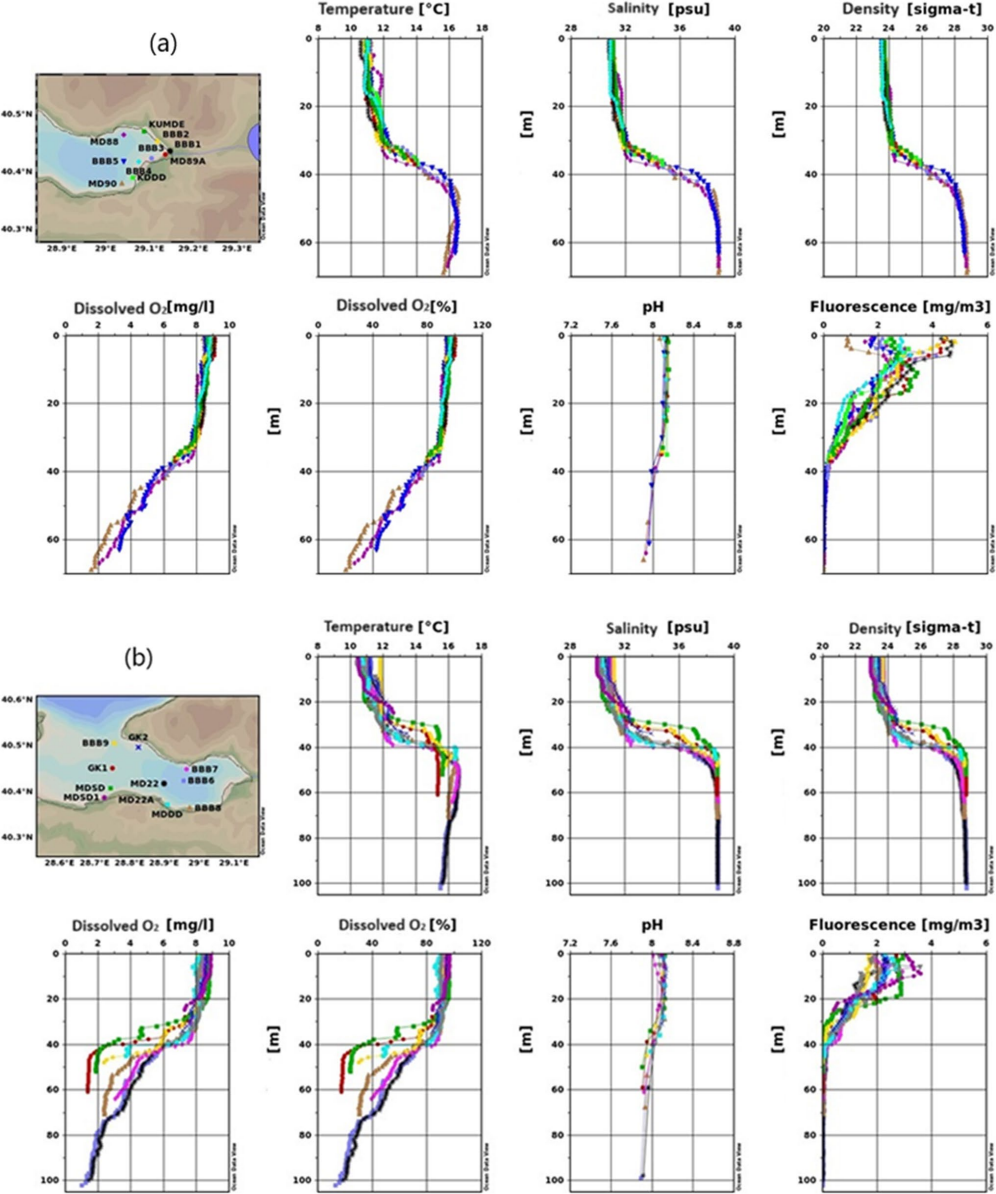

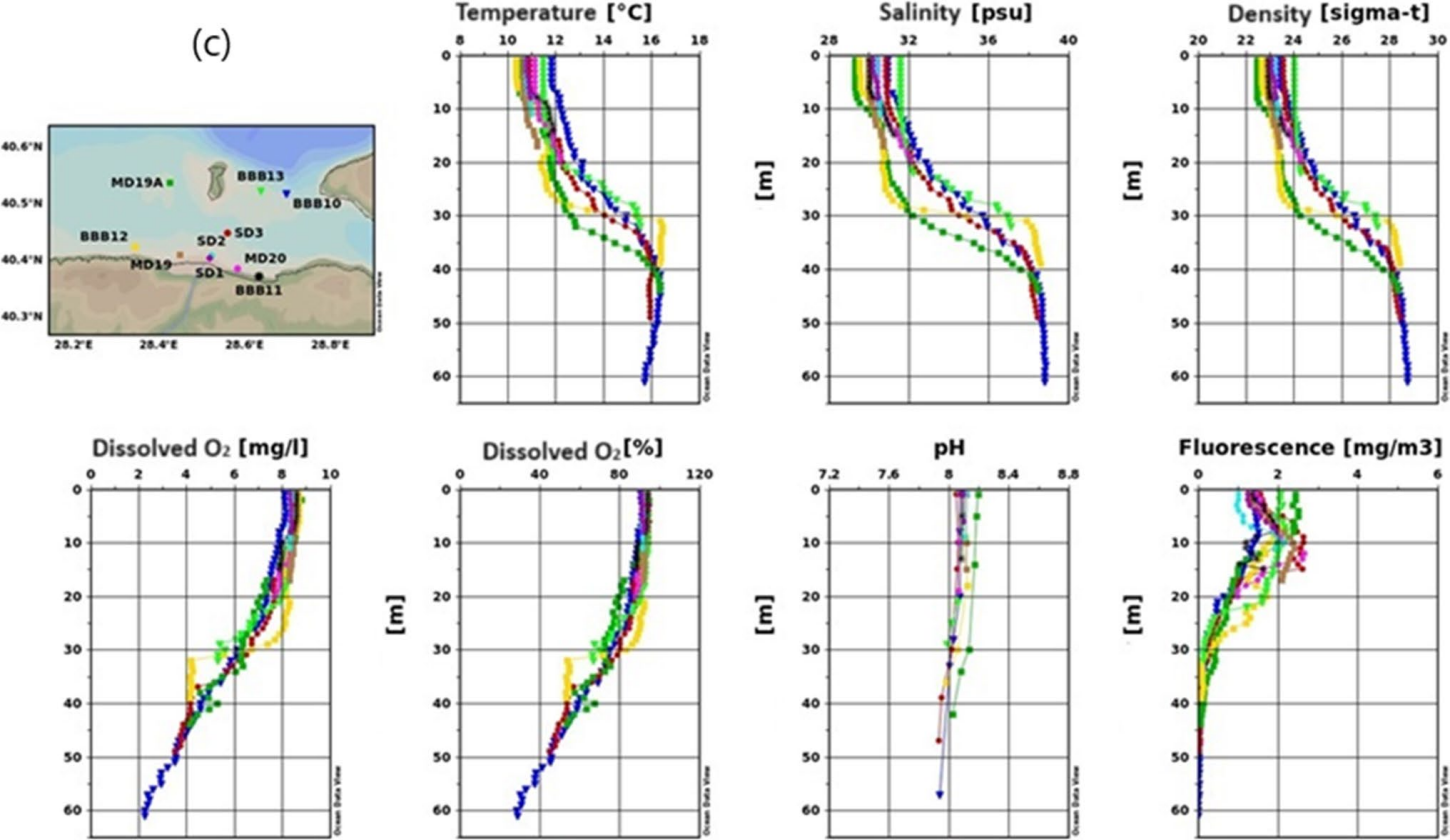
Results of temperature, salinity, density, dissolved oxygen, pH, and fluorescence measurement for the 2022 winter season (**a** inner basin of Gemlik Bay, **b** Gemlik Bay, **c** Susurluk river)

Figure 2 shows changeability in the spatial distribution of water salinity, temperature, pH, sea density, fluorescence, and DO through the studied area.

##### Water temperature

Water temperature plays an essential role in both evaluating water quality and understanding aquatic ecosystems. It has a substantial impact on a wide range of chemical and biological processes, which in turn affects the distribution and environmental circumstances of aquatic life (Ustaoglu and Tepe 2019).

Figure 2a indicates that in the Inner Basin of the Gemlik Gulf during the winter season, surface temperatures average around 11 °C, with an increase in temperature observed below 25 m. The temperature slightly exceeds 16 °C around 45 m and then decreases slightly below 16 °C toward 70 m. Figure 2b shows surface temperatures ranging between 10.5 and 12 °C, with a sudden increase between 30 and 40 m, stabilizing around 16 °C below 40 m. The observed temperature variation could be related to the mixing of freshwater and the varying intensity of water currents (Singh et al. 2022). Figure 2c describes the region where the Susurluk River discharges, with surface temperatures ranging between 10 and 12 °C. Notably, stations like MDSD, BBB9, and GK1 exhibit warmer and saltier water masses between 30 and 40 m, likely due to upwelling.

In all figures, the winter season is characterized by cooler surface temperatures, with increasing temperatures at greater depths, followed by a slight decrease. The temperature increase between 25 and 40 m is significant and likely related to water mixing and freshwater influx. Upwelling effects in certain areas lead to more pronounced temperature differences.

##### Salinity

Salinity is observed to be lower at the surface and increases with depth in all three figures. Figure 2a reports surface salinity around 31 psu, which is higher than in the summer due to wind-driven mixing. Salinity increases significantly at around 40 m, converging to the lower layer’s salinity of 38.7 psu. Several factors influence coastal water salinity, including water shallowness, tide patterns, low rainfall, high evaporation, and the region’s extreme aridity, in addition to the neritic water effect (Geng et al. 2016).

Figure 2b shows surface salinity between 30 and 31 psu, increasing with depth and stabilizing below 50 m. Figure 2c mentions that in the Susurluk River discharge region, surface salinity varies between 29 and 32 psu, with noticeable differences at 30 m. Stations near Armutlu coasts show distinct salinity variations, likely due to upwelling.

A significant salinity increase is noted between 30 and 40 m, likely due to mixing processes and the upward movement of deeper, saltier water masses. The Susurluk River discharge region shows more pronounced salinity differences, influenced by upwelling currents.

##### Dissolved oxygen

Dissolved oxygen (DO) levels are high near the surface and decrease with depth across all figures. The high concentration of DO in coastal water may be caused by the photosynthetic process, the combined impacts of heavy rainfall, and a rise in wind speed, which results in a higher entry of freshwater (Comfort et al. 2019; Hammer et al. 2019). This trend is likely due to the influence of temperature and salinity on oxygen solubility. Figure 2a shows that DO levels range between 8 and 9 mg/L down to 30 m, with a decrease toward the sea floor, reaching 2 mg/L at 70 m. When approaching 70 m in depth, it had dropped to 2 mg L^−1^. The temperature and salinity of the water also have an effect on oxygen solubility (Arnott et al. 2021). Figure 2b indicates that surface DO levels are between 8 and 9 mg L^−1^, decreasing to 1 mg L^−1^ in the Burgaz Çukuru. Figure 2c suggests that in the Susurluk River discharge region, DO levels between 30 and 40 m differ from other areas, dropping to 2 mg L^−1^ at 60 m. The Susurluk River discharge region shows distinct DO variations, reflecting the dynamic nature of the water movement in that area. The lower DO levels in the Burgaz Çukuru suggest potential anoxic or hypoxic conditions.

##### Fluorescence (phytoplankton density)

In marine environments, fluorescence measurements offer a potent tool for comprehending, observing, and evaluating a range of biological and chemical properties. Fluorescence measurements show non-uniformity across all stations, much like other parameters. Figure 2a indicates that surface fluorescence values vary, with the lowest at MD90 and the highest at BBB2, BBB1, and MD89 A, reaching values close to 5 mg/m^3^. Figure 2b shows that surface fluorescence reaches a maximum of 3.75 mg/m^3^, rapidly decreasing below 20 m and becoming zero below 40 m. Figure 2c describes the Susurluk River discharge region, where surface fluorescence values are lower than in other regions, with a maximum of around 2.5 mg/m^3^. Fluorescence drops to zero below 30 m.

Fluorescence values are higher at the surface and decrease with depth across all figures, indicating higher phytoplankton density near the surface. The Susurluk River discharge region shows lower fluorescence levels, suggesting lower biological productivity or different environmental conditions in that area. The high fluorescence measurements in the seas generally indicate an increase in the density of phytoplankton or other photosynthetic organisms. This situation can indicate an increase in biological productivity in the marine ecosystem or a high concentration of phytoplankton in a specific area. Phytoplankton are microscopic plant-like organisms that produce energy through photosynthesis by utilizing sunlight. Apart from the surface differences, the change towards deeper layers is as follows.

##### pH

The pH level is another critical chemical parameter that has important biological implications in natural water systems. Variations in pH can affect the toxicity of specific compounds present in water (Boyd et al. 2016). While the seawater pH is around 8.1 in the upper layer, it has fallen below 8 pH below 40 m due to the decrease in oxygen. pH decreases with depth, which can be attributed to the reduction in oxygen levels and the potential increase in dissolved CO_2_ or other acidic compounds.

##### Density

Density, influenced by temperature and salinity, plays a crucial role in water column stratification and mixing. Figure 2a shows that the surface layer in the Inner Basin of the Gemlik Gulf has relatively low density due to colder temperatures, while density increases with depth as the water becomes warmer and saltier, particularly below 40 m. Figure 2b demonstrates that surface waters are less dense, with increasing density at greater depths, consistent with the warming and salting of deeper water layers. Figure 2c indicates that in the Susurluk River discharge region, surface waters are less dense, with higher density observed below 30 m. The upwelling near Armutlu coasts contributes to significant density differences.

#### Spring season

**Fig. 3:**
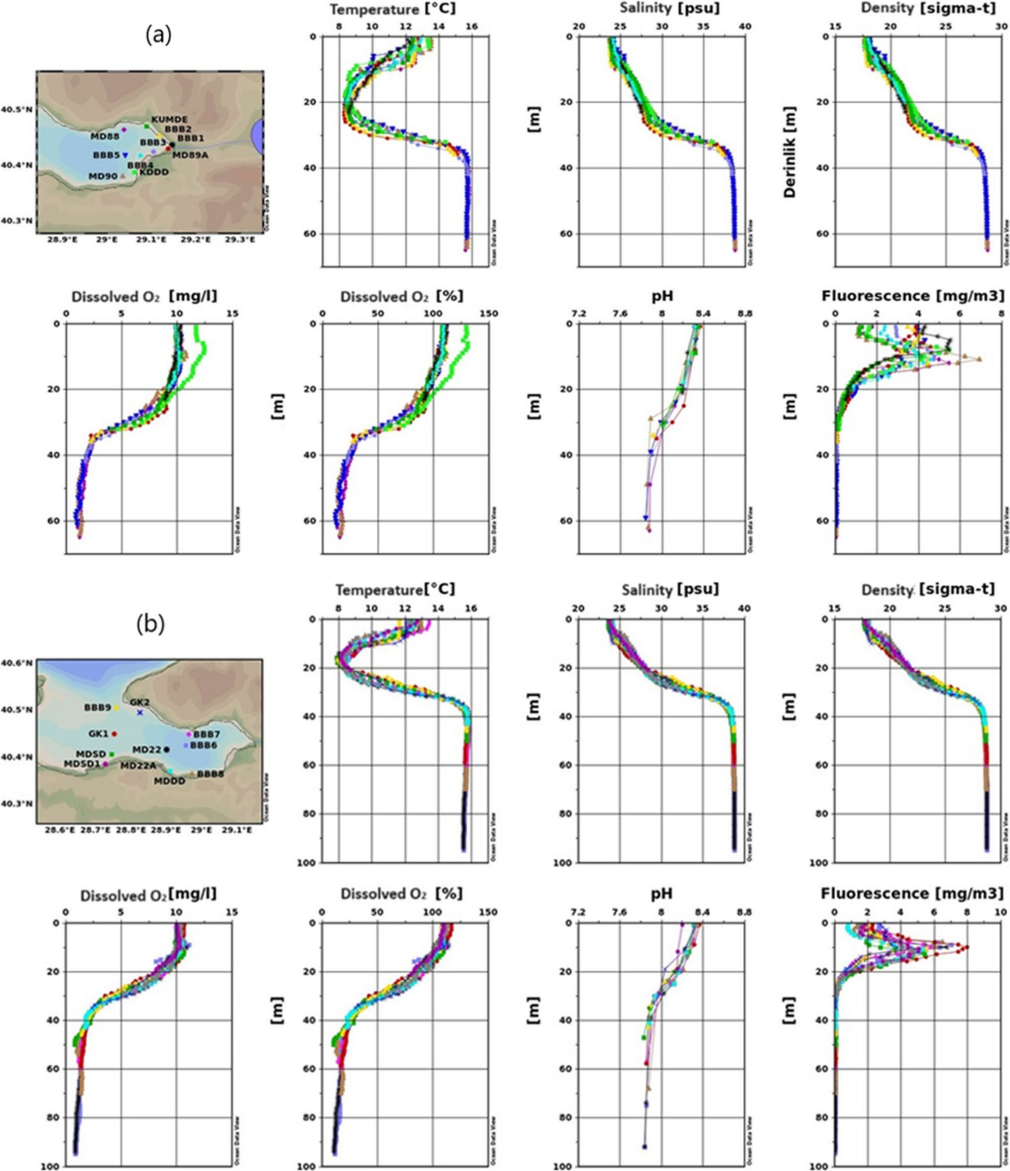

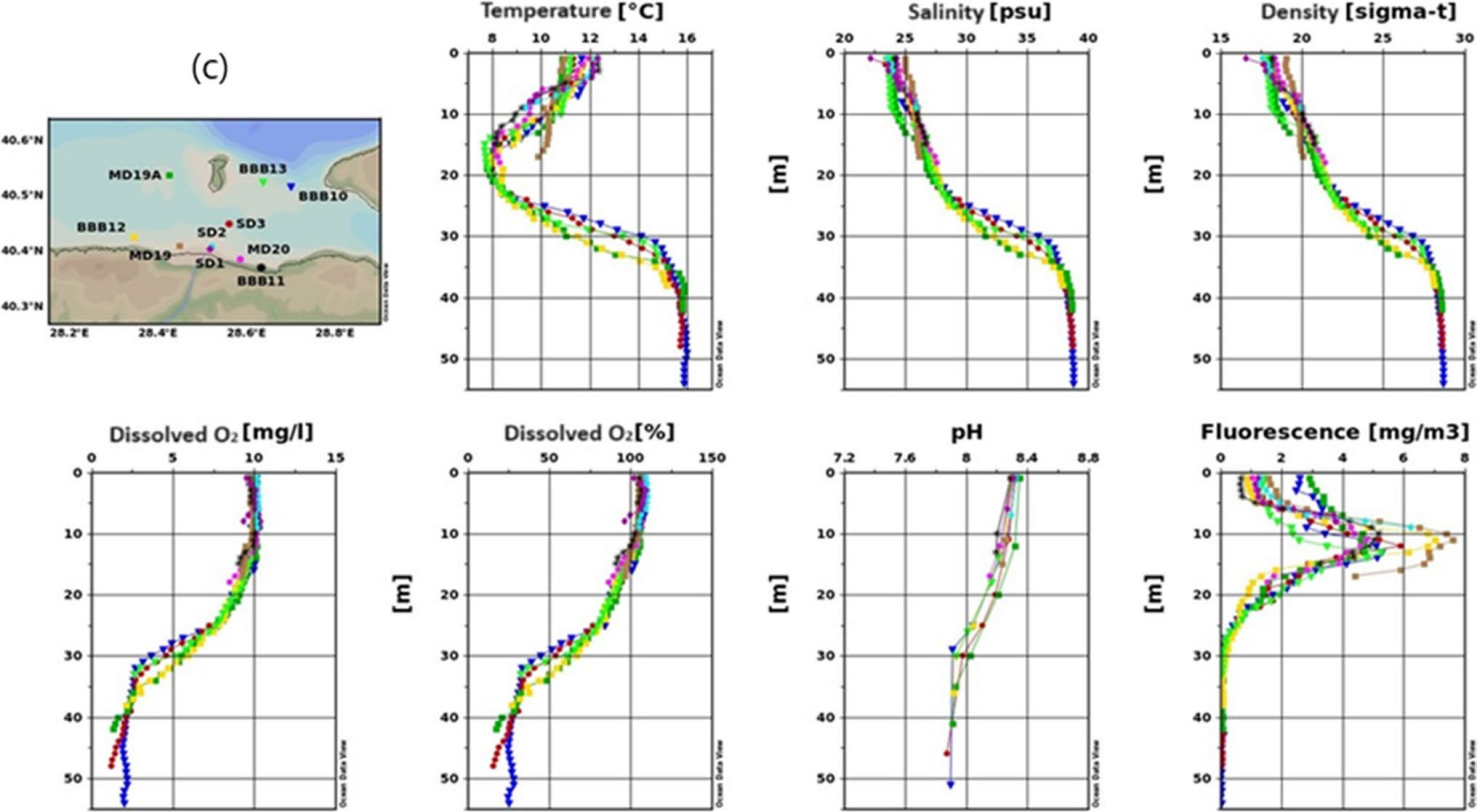
Results of temperature, salinity, density, dissolved oxygen, pH, and fluorescence measurement for the 2022 spring season (**a** inner basin of Gemlik Bay, **b** Gemlik Bay, **c** Susurluk river)

Figure 3 shows changeability in the spatial distribution of water salinity, temperature, pH, sea density and DO through the studied area for spring season.

##### Temperature

When evaluating the water quality parameters of Gemlik Bay during the spring season of 2022, it is observed that surface warming has occurred (Fig. 3). Figure 3a shows surface temperatures during the spring season range between 12 and 14 °C. A cold water mass from the winter season is present at around 20 m, with temperatures between 8 and 9 °C. Below 40 m, temperatures stabilize around 15.5 °C. Figure 3b indicates surface temperatures vary slightly, with lower temperatures at the BBB9 station and higher at the BBB7 station. Temperatures decrease to around 8 °C at 15 m, then increase rapidly to 16 °C at 40 m before slightly decreasing again. Figure 3c mentions that surface temperatures range between 11 and 12.5 °C, decreasing to below 8 °C at 15–20 m. At the MD19 station, a temperature anomaly is observed at and below 10 m, possibly due to local influences like river inflow. Temperatures then increase to 16 °C at 40 m.

Generally, surface temperatures are within a similar range (11–14 °C). A cold water mass is consistently observed at around 15–20 m, likely a remnant of the winter season. The temperature anomaly at the MD19 station in Fig. 3c suggests localized factors influencing the temperature profile, while the overall trend shows increasing temperatures with depth, stabilizing around 16 °C.

##### Salinity

Salinity values are similar at all stations, approximately 24 psu on the surface, and have decreased compared to the winter season, attributed to the reduction in stormy periods (Balcı et al. 2012; Singh et al. 2022). Salinities have increased in the range of 10–30 m, with a sudden rise occurring between 30 and 35 m. In deeper layers, the lower layer salinity (38.7 psu) is observed. A negative salinity anomaly is detected at 1 m at the SD1 station, likely related to freshwater input from the Susurluk River.

##### Dissolved oxygen

Surface DO levels are generally around 10 mg/L at most stations, except for the KDDD station, where it rises to 12.5 mg L^−1^. Below 20 m, DO levels decrease, reaching 1–2 mg L^−1^ below 35 m. Figure 3c shows a more rapid decline in DO levels, likely due to increased organic matter and subsequent oxygen consumption near the river discharge area. Figure 3a shows a slight anomaly at the KDDD station with higher surface DO levels, which could be due to localized environmental conditions.

##### Fluorescence

Fluorescence quantity varies on the surface between 1 and 4 mg/m^3^, with the highest values observed at stations located in the inner part, such as BBB1, MD89 A, and BBB2.

Figure 3a shows fluorescence peaks around 10 m and drops to zero below 40 m.

According to Fig. 3b, fluorescence values drop to zero at all stations below 20 m.

Figure 3c indicates surface fluorescence varies between 0.5 and 3 mg/m^3^, with the highest value at MD19 A. Peak values are observed around 10 m at MD19 and BBB12, approaching 8 mg/m^3^. Fluorescence drops to zero below 25 m.

Fluorescence values, indicative of phytoplankton density, peak near the surface and around 10 m in all figures, with the highest readings at stations influenced by nutrient inputs, such as those near the Susurluk River in Fig. 3c. The rapid decrease to zero in deeper layers across all figures suggests limited light penetration and reduced biological activity at greater depths. The highest fluorescence values in Fig. 3b and c are similar, indicating areas of high biological productivity, likely due to localized nutrient enrichment.

#### Summer season

During the summer season, water quality parameters such as temperature, salinity, dissolved oxygen, and fluorescence exhibit a consistent pattern with minor variations due to localized influences such as river discharge and seasonal effects. The thermal structure across the bay remains stable, with warm surface waters and a persistent cold water mass at depth. Salinity increases sharply in the intermediate layer, while dissolved oxygen decreases with depth, showing signs of localized oxygen depletion in certain areas. Fluorescence levels indicate active primary production, particularly influenced by river inputs.

##### Temperature

**Fig. 4:**
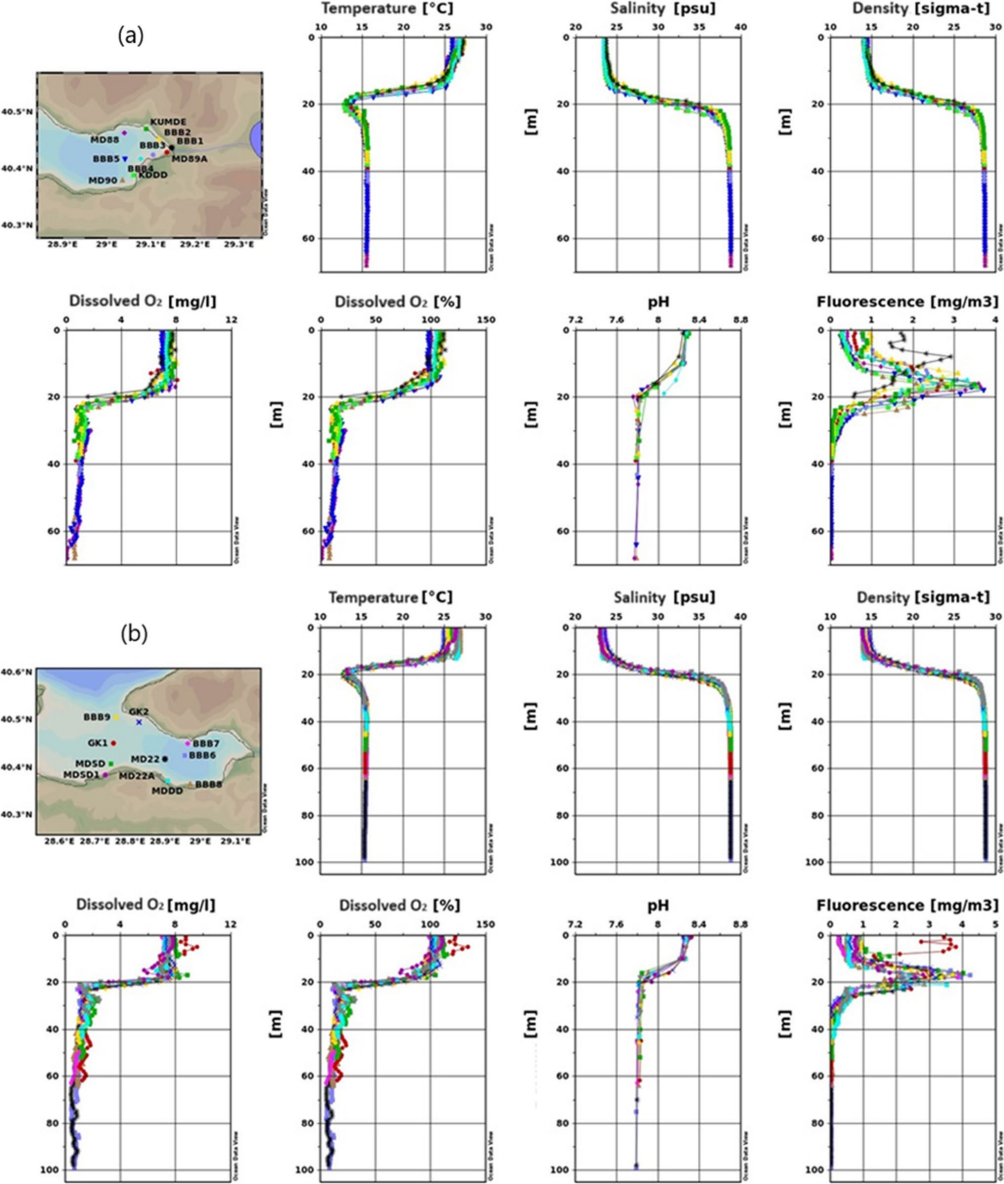

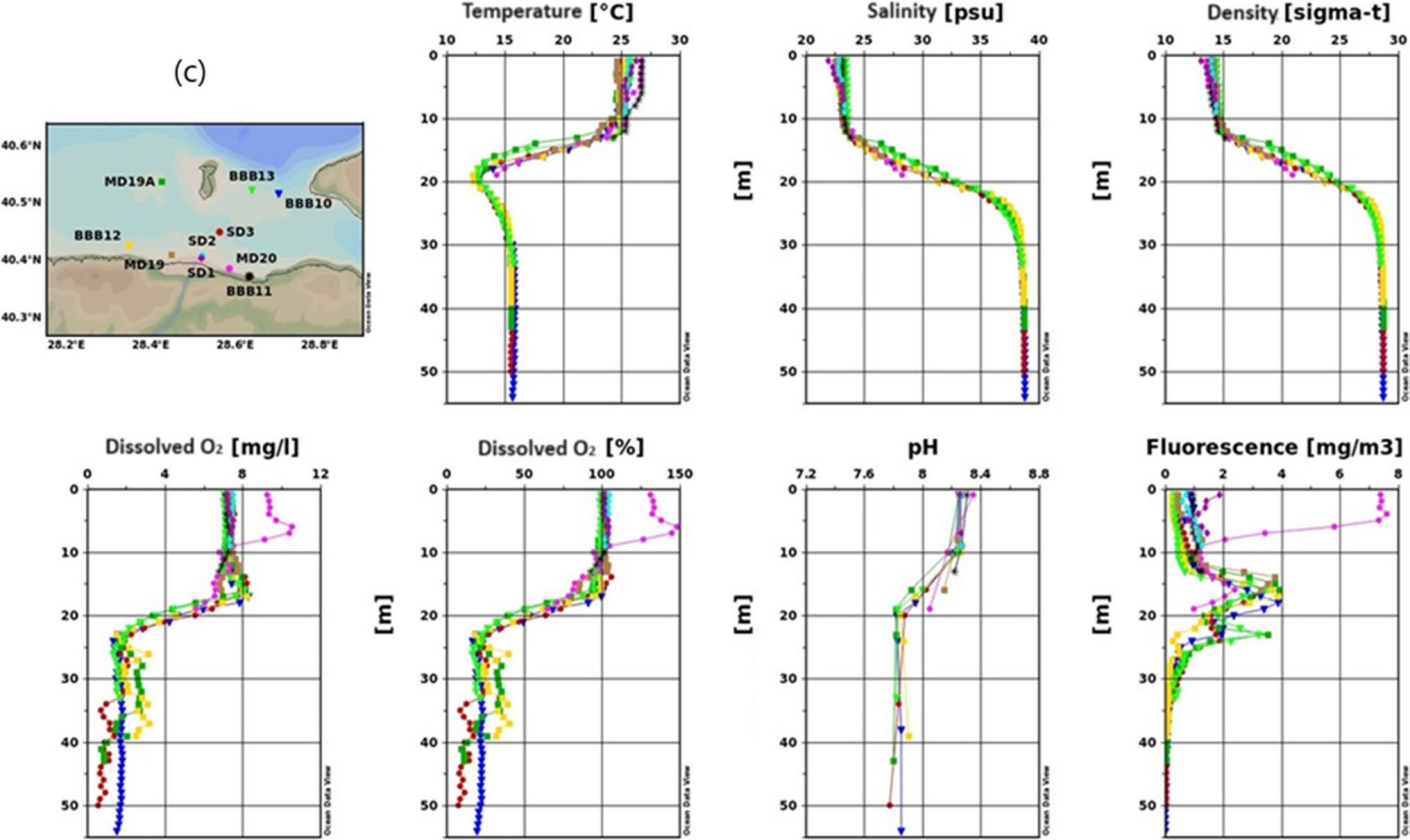
Results of temperature, salinity, density, dissolved oxygen, pH, and fluorescence measurement for the 2022 summer season (**a**- inner basin of Gemlik Bay, **b**-Gemlik Bay, **c**-Susurluk river)

As observed in Fig. 4, due to the summer season, a warm surface water mass is present. Temperatures have ranged between 25 and 27 °C, relatively high for seawater. The mixed layer is 15 m thick, and below it, at a depth of 20 m, a cold water mass from the winter season is observed. Deeper down, the characteristics of the lower layer prevail, with temperatures around 15 °C.

The similarities indicate a consistent thermal structure throughout the Gemlik Bay during the summer season, with the Susurluk River discharge area showing a comparable temperature profile to the rest of the bay.

##### Salinity

Salinity was measured around 23–24 psu on the surface, showing a sudden increase in the intermediate layer and stabilizing in the lower layer (Fig. 4). Salinity profiles show similar patterns across all figures, with surface salinity slightly lower in the Susurluk River (Fig. 4c).

The sharp increase in salinity in the intermediate layer and stabilization in the lower layer is consistent across the bay. The lower surface salinity in the Susurluk River area suggests the influence of freshwater input from the river.

##### Dissolved oxygen

In the water profile, oxygen levels were measured between 7 and 8 mg L^−1^ on the surface and in the mixed layer (Fig. 4). In the intermediate layer, there was a sudden decrease trend and in the lower layer, it remained below the critical level. However, at the MD88 station and at the bottom, oxygen levels were observed to drop to zero.

According to Fig. 4c, oxygen levels dropped below 4 mg L^−1^ at depths greater than 25 m, with significant variations observed at the SD3 and BBB10 stations, likely influenced by river input. This indicates the presence of an oxygen-consuming pressure in this region.

##### Fluorescence

Fluorescence levels were concentrated in the mid-water column, particularly between 15 and 20 m, across all figures. Figure 4c shows slightly higher fluorescence values, especially at MD20, likely due to nutrient input from the Susurluk River, promoting higher primary production. The variations in surface fluorescence, particularly at the BBB1 station in Fig. 4a and b, suggest differences in local conditions or measurement timing.

#### Autumn season

##### Temperature

**Fig. 5:**
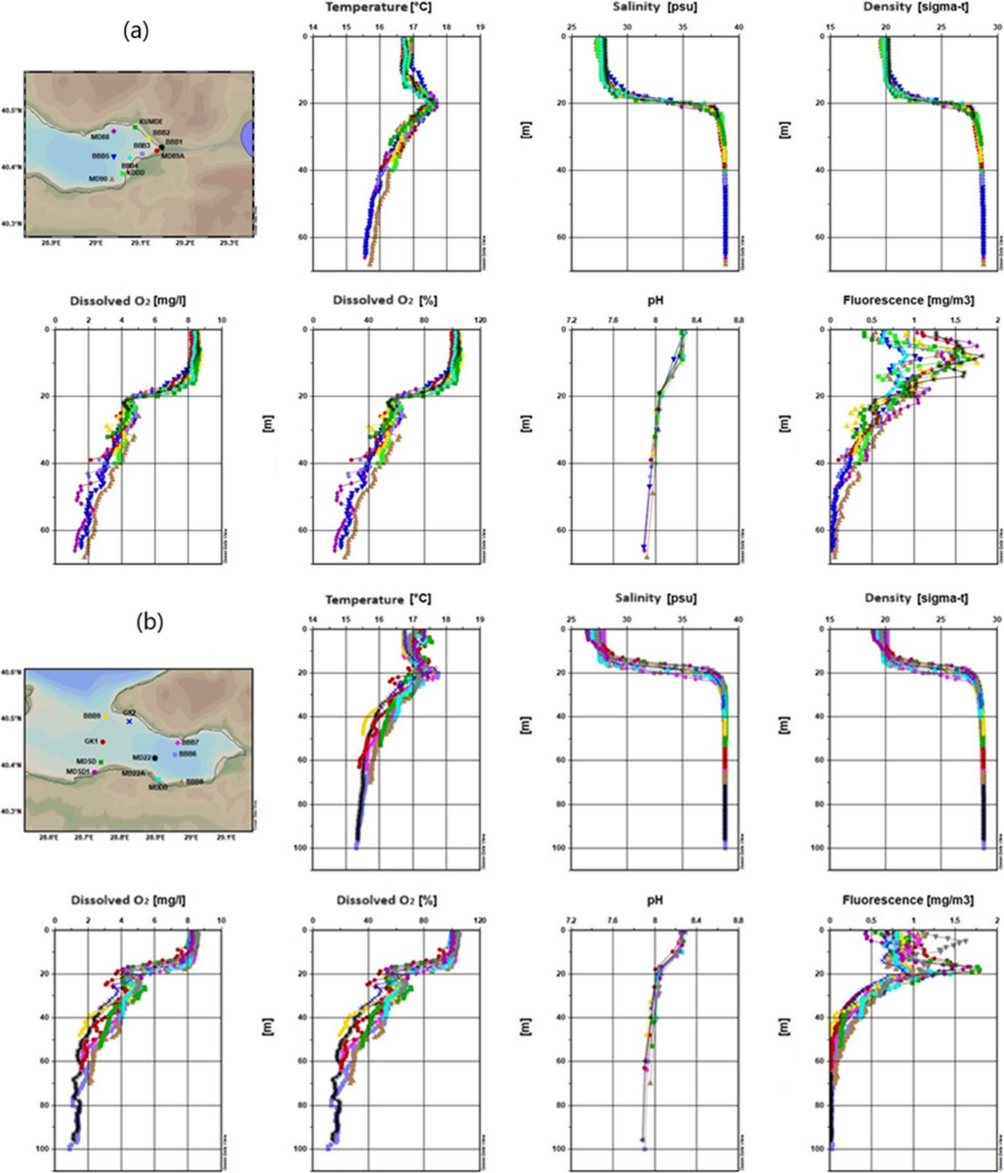

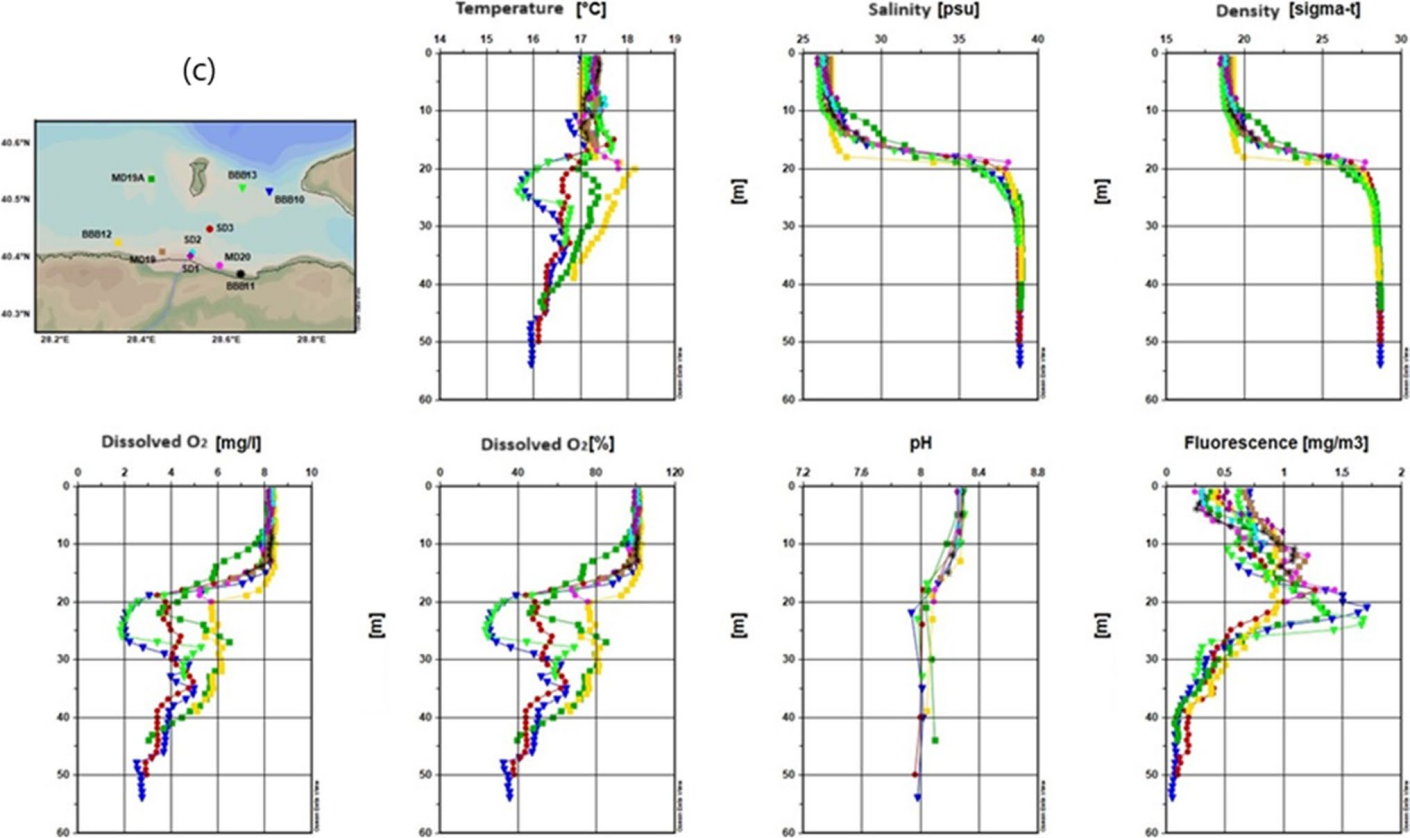
Results of temperature, salinity, density, dissolved oxygen, pH, and fluorescence measurement for the 2022 autumn season in the Susurluk River (**a**- inner basin of Gemlik Bay, **b**-Gemlik Bay, **c**-Susurluk river)

Due to the autumn season, cooling has been observed on the surface. Therefore, while surface temperatures are in the range of 16.5–17.0 °C, a measurement of 17.5 °C was recorded at a depth of 20 m. Further down, it has decreased and approached 15.5 °C (Fig. 5).

In the area where the Susurluk River discharges, surface temperature was 17.5 °C. Below 15 m, temperatures varied across stations; at BBB13 and BBB10, temperatures decreased, while at BBB12, they increased. This variation suggests the approach of a cold water mass from the North (Fig. 5c). The approach of this colder water mass, particularly in Fig. 5b, highlights the transitional nature of the season, with external water masses influencing the thermal structure of the bay.

##### Salinity

Salinity levels show a general increase on the surface across all figures, reflecting the effects of stronger winds and seasonal changes. Figure 5a and b indicates consistent salinity structures with stable lower layers. Surface salinity varied between 26 and 28 psu, also showing an increase compared to the summer season. The intermediate layer depth varied by station, but a consistent sublayer was observed below 30 m. In Fig. 5c, the lack of an average salinity value between 15 and 30 m suggests significant variability, potentially due to the influence of river discharge and external water masses.

##### Dissolved oxygen

Dissolved oxygen levels exhibit a downward trend with depth across all figures, consistent with seasonal cooling and the influence of external water masses. Figure 5a shows a slight decline in oxygen levels, while Fig. 5b highlights the significant impact of incoming water masses, resulting in lower oxygen levels at depth. Oxygen levels continued to decrease in deeper layers, reaching around 1 mg/L at the bottom, influenced by water masses from outside the gulf. The lowest value of 1 mg/L recorded at the MD88 station. Primary production has moved up to the upper layer.

Figure 5c indicates even greater variability in oxygen levels, particularly at 25 m, due to the dynamic environment near the Susurluk River discharge. The presence of a cold water mass, low in oxygen, contributed to these lower levels.

##### Fluorescence

Fluorescence levels generally decreased across all figures, reflecting the seasonal reduction in primary production as the water cools. Figure 5a and b shows similar trends, with fluorescence concentrated in the upper 20 m. Figure 5c highlights the influence of river discharge on fluorescence levels, with higher values near the surface, likely due to nutrient inputs. The overall reduction in fluorescence suggests a decline in phytoplankton activity as the season progresses. The maximum amount was determined to be 1.7 mg/m^3^.

Overall, during the autumn season, salinity increases are observed across the bay, while dissolved oxygen levels decrease with depth, particularly influenced by external water masses. Fluorescence levels, indicative of primary production, have generally decreased, reflecting the seasonal transition and the impact of external inputs such as the Susurluk River.

### Data analysis

The strength of correlation among water quality parameters was assessed using the Pearson Correlation Index, and the results are summarized in Table 1. Correlation magnitude was interpreted as follows: coefficients between 0.9 and 1 indicated a very high relationship, 0.7 to 0.89 signified a high relationship, 0.5 to 0.69 indicated a moderate relationship, 0.26 to 0.49 suggested a weak relationship, and 0 to 0.25 represented a very weak relationship. Additionally, negative values indicated an inverse relationship, where an increase in one parameter corresponded to a decrease in the other. For example, a moderate negative correlation was observed between salinity and temperature (*r* = − 0.599; *p* < 0.01). In addition, weak negative correlations were found between DO and NH_4_-N, DO and TN (*r* = − 0.289 and − 0.384; *p* < 0.01).

The results indicate that there is a significant negative correlation between temperature and dissolved oxygen (DO), which is consistent with the accepted notion that oxygen solubility increases in cooler water (Post et al. 2018; Vega et al. 1998). Furthermore, there were notable inverse relationships between DO and nutrients such NH_4_-N, TN, and TP. Higher concentrations of organic matter result from increasing nutrient levels, which also promote respiration and organic matter decomposition, which lower DO levels (Chau and Jiang 2002; Varol and Li 2017; Varol 2020). Strong positive correlations between NH_4_-N and TN show that these variables have comparable sources, with untreated household sewage and agricultural runoff being identified as the main causes of pollution for these components in the sea. Temperature and Chl-a had a strong negative association, but salinity, TN, and NH_4_-N showed positive correlations. TP showed weakly positive correlation (*p* < 0.05).

**Fig. 6:**
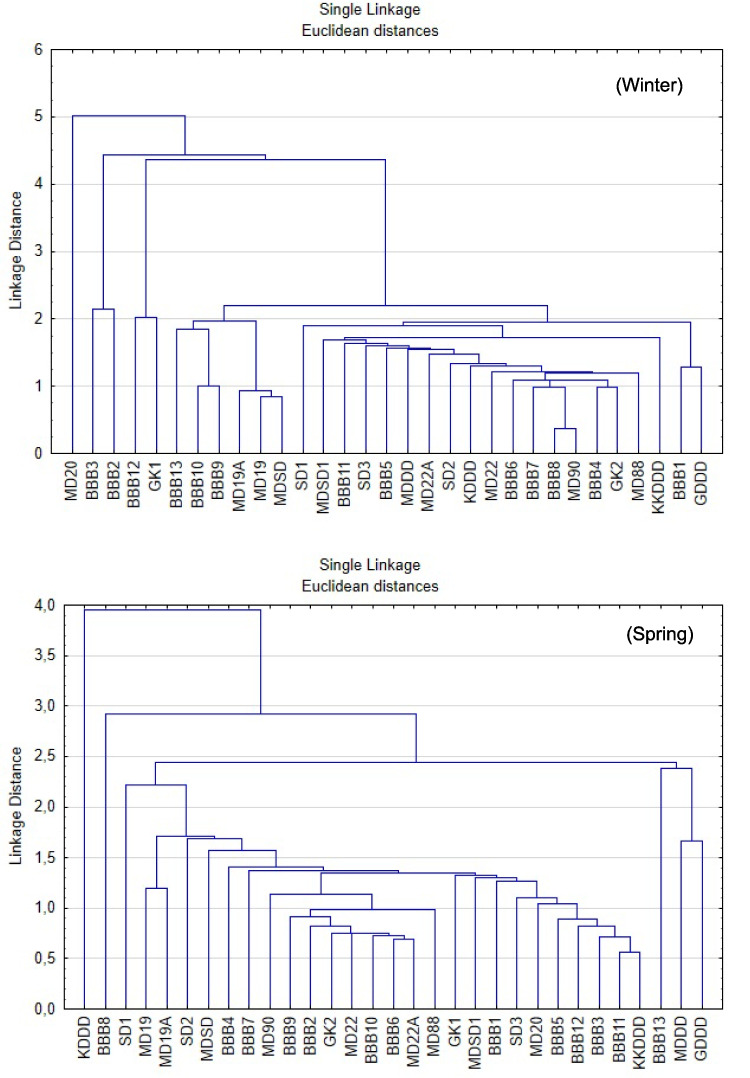

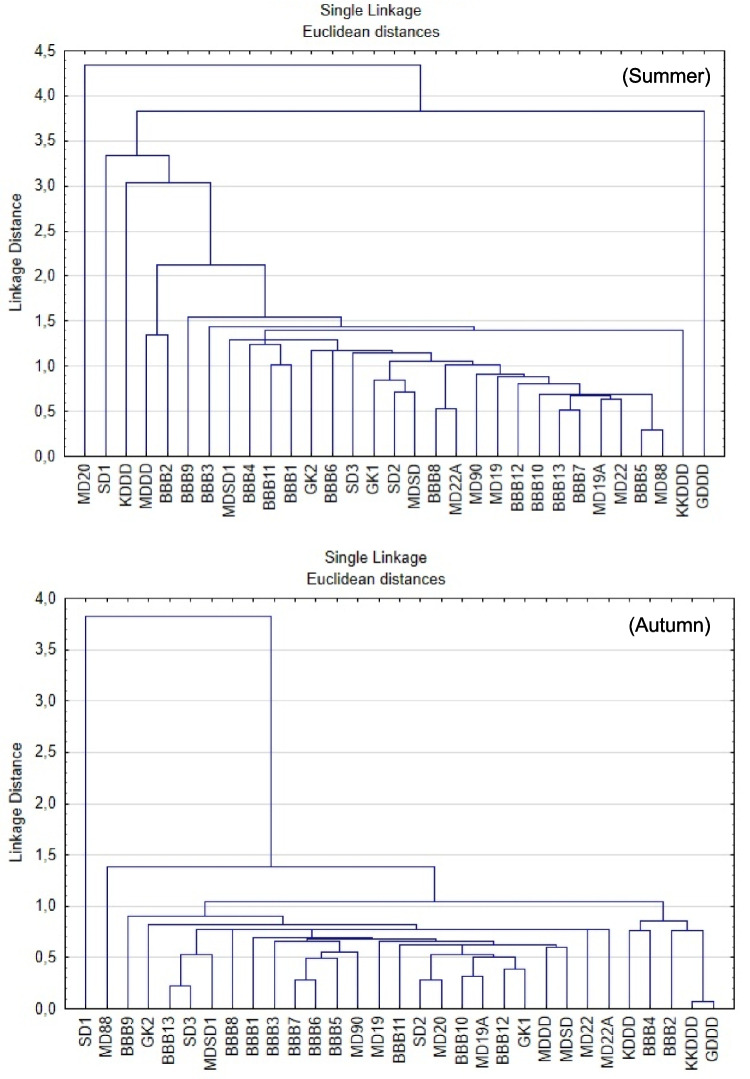
Cluster analysis of the sampling different stations for environmental status on Euclidean distance

Cluster analysis (CA) is widely used in water quality studies to group sampling sites into clusters/regions based on their similar characteristics and to contribute to spatial discriminant analysis (Li et al. 2018; Varol 2020). Applying a hierarchical clustering technique for stations, the dendrogram is depicted in Fig. 6.

The group average assemblages of the sampling points from Euclidean distance indicated similar clustering based on the environmental parameters. Seasonal variations among the stations were clearly observed. In winter season, stations were mostly similar, except for KKDDD and SD3. In spring, heterogeneity increased due to seasonal transition, and stations such as GDDD and MDDD were clearly separated from the others. In summer, differentiation peaked, particularly at GDDD, MD20, and SD1, likely due to increased anthropogenic pressures, higher temperature, and reduced water circulation. By autumn, the system showed partial recovery; however, SD1 and MD88 remained distinct, suggesting ongoing pollution stress.

Overall, the MD20, SD1, and GDDD stations have emerged as high-risk areas. An analysis of the station locations reveals that they are positioned near the discharge points of the Nilüfer and Susurluk rivers, as well as the deep-sea discharge site of Gemlik-Kurşunlu. In contrast, GK1 and BBB12 stations remained stable and less-impacted areas throughout the year.

In order to identify differences among stations for each season and nutrients parameter, one-way ANOVA followed by Tukey’s HSD post-hoc test was applied. During the winter season, no statistically significant differences were observed among stations for NOx and DO parameters. In contrast, a significant spatial variation was detected for total nitrogen (TN). In particular, the MD20 station exhibited significantly different TN concentrations compared to several other stations, including KKDDD, GK1, MD19, and BBB12 (*p* < 0.05), suggesting the possible influence of localized nitrogen inputs or retention processes at this site.

For TP, the SD3 station showed statistically significant differences compared to BBB6–10, GK2, MDDD, and KDDD, indicating a distinct nutrient dynamic in this region (*p* < 0.05). Furthermore, regarding chlorophyll-a, the BBB10 and MD89 A stations presented significantly different (*p* < 0.05) values from the other stations, potentially reflecting site-specific eutrophication levels or variations in primary productivity.

Although NH₄-N concentrations were relatively similar across most stations, the SD1 station showed significantly different values when compared to stations such as MDSD, MD19, MD20, GK1, and BBB12. Likewise, the MD22 station significantly differed from MDSD, MDSD1 and SD3, which may indicate the presence of a unique nitrogen source or removal mechanism at that location (*p* < 0.05).

During spring, summer, and autumn, there were no statistically significant differences in DO, NOx, and chlorophyll-a concentrations among stations, indicating relatively consistent distribution of these parameters. However, NH₄-N showed statistically significant spatial variation across all seasons. In particular, the MD89 A station had significantly different NH₄-N levels, especially when compared to SD3, MD88, GK1, BBB5, and BBB6 (*p* < 0.05).

For TP, the KDDD station was found to be significantly different from SD3, BBB2, and BBB3 during the spring season (*p* < 0.05). These findings suggest that, while nutrient and oxygen-related parameters were generally consistent among stations in spring, localized nutrient inputs or retention processes at stations such as MD89 A and KDDD may have contributed to spatial heterogeneity in NH₄-N and TP levels.

Finally, during the summer season, MD22 and BBB9 stations exhibited significantly different TN concentrations compared to the other stations (*p* < 0.05), indicating potential site-specific nitrogen enrichment in these locations.

### Trophic status and water quality class

In Turkey, the trophic status and water quality class are assessed based on various parameters to determine the overall health and nutrient levels in aquatic ecosystems. The classification often involves factors such as nutrient concentrations, chlorophyll-a levels, and transparency. The trophic status and water quality classification presented in the Supplementary Section are typically categorized into classes such as oligotrophic, mesotrophic, eutrophic, and hypereutrophic (Table S3). These classes provide insights into the nutrient enrichment and overall ecological balance of water bodies, helping in the evaluation of potential environmental impacts.

The eutrophication criteria for the coastal waters of the Sea of Marmara include various parameters used to determine water quality and assess the health of the ecosystem in the lake. Eutrophication is a condition in aquatic systems where excessive nutrient loading results in rapid growth of vegetation.

**Fig. 7:**
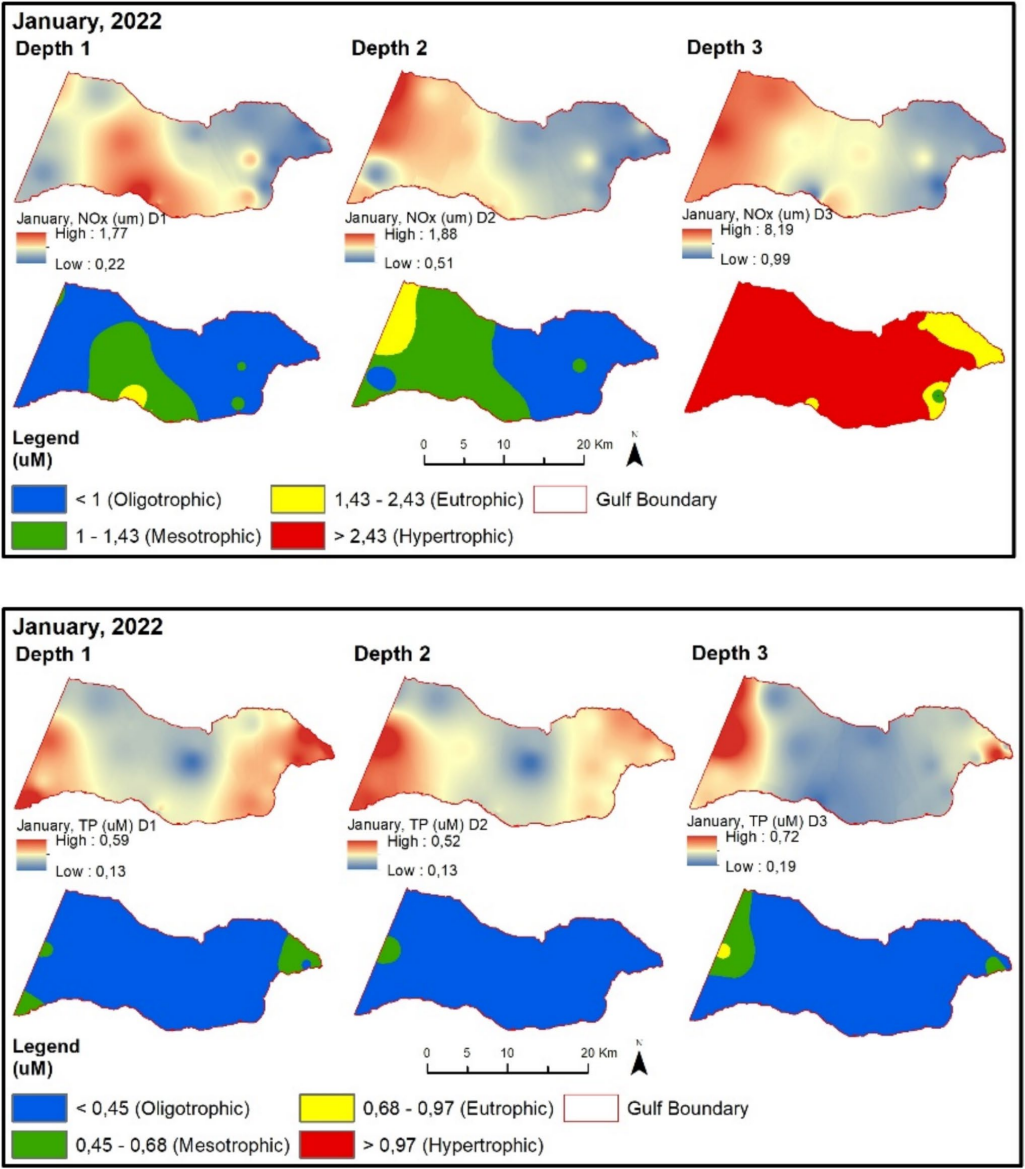

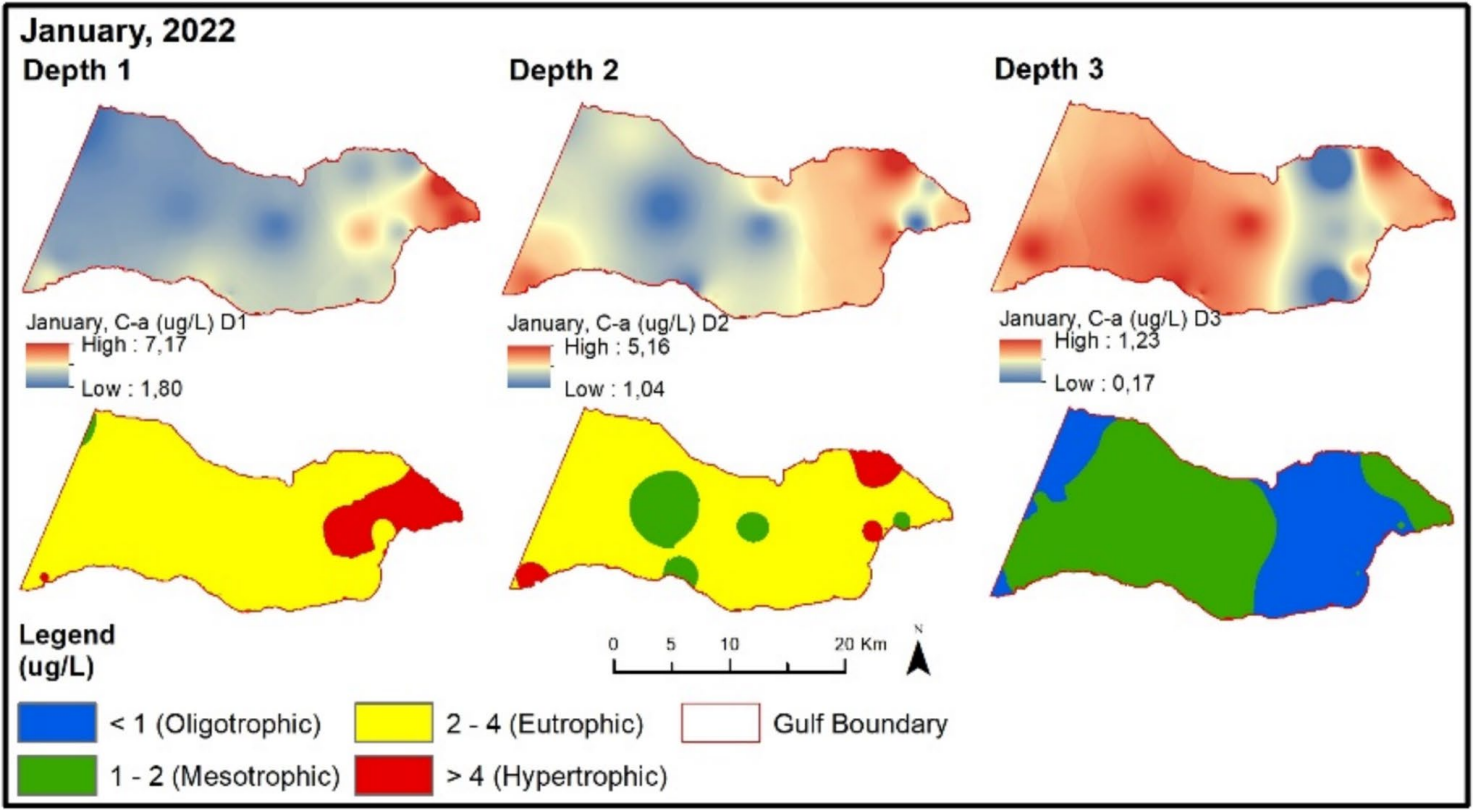
Evaluation of depth-dependent eutrophication criteria for the Gemlik Gulf in January 2022 (Depth 1: 0–5 m; Depth 2: 5–20 m; Depth 3: > 20 m)

According to the Surface Water Pollution Control Regulation for the year 2022, an assessment reveals that in the January period, the turbidity depth is higher at points outside the gulf and not close to the coast (Table 2). During January, NOx values are generally at oligotrophic levels (Fig. 7). It is observed that points with high NOx values in surface water are located near the coast. In the same period, points with high TP values are higher in locations influenced by river inputs compared to other points.

**Table 2:**
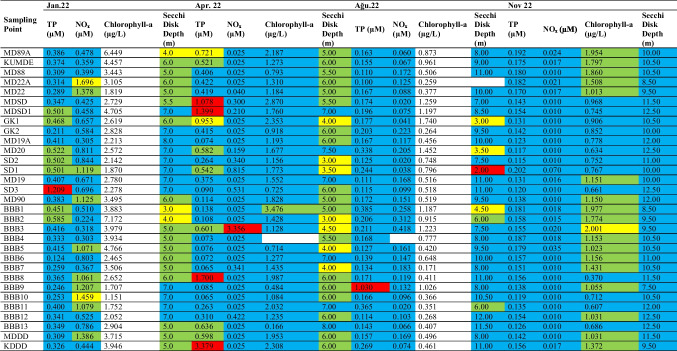
Evaluation of the Gemlik Gulf according to surface water quality management regulation

**Fig. 8:**
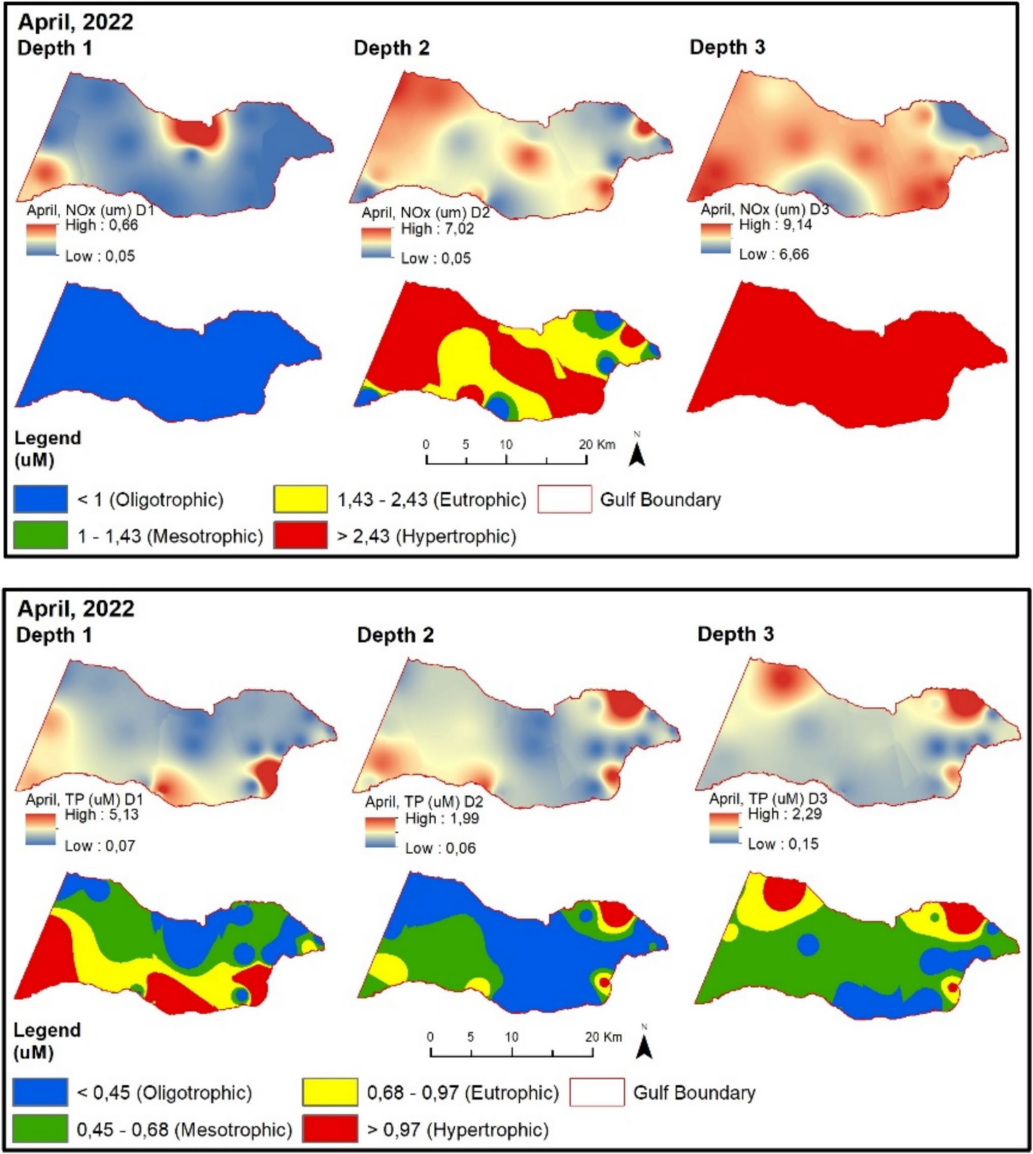

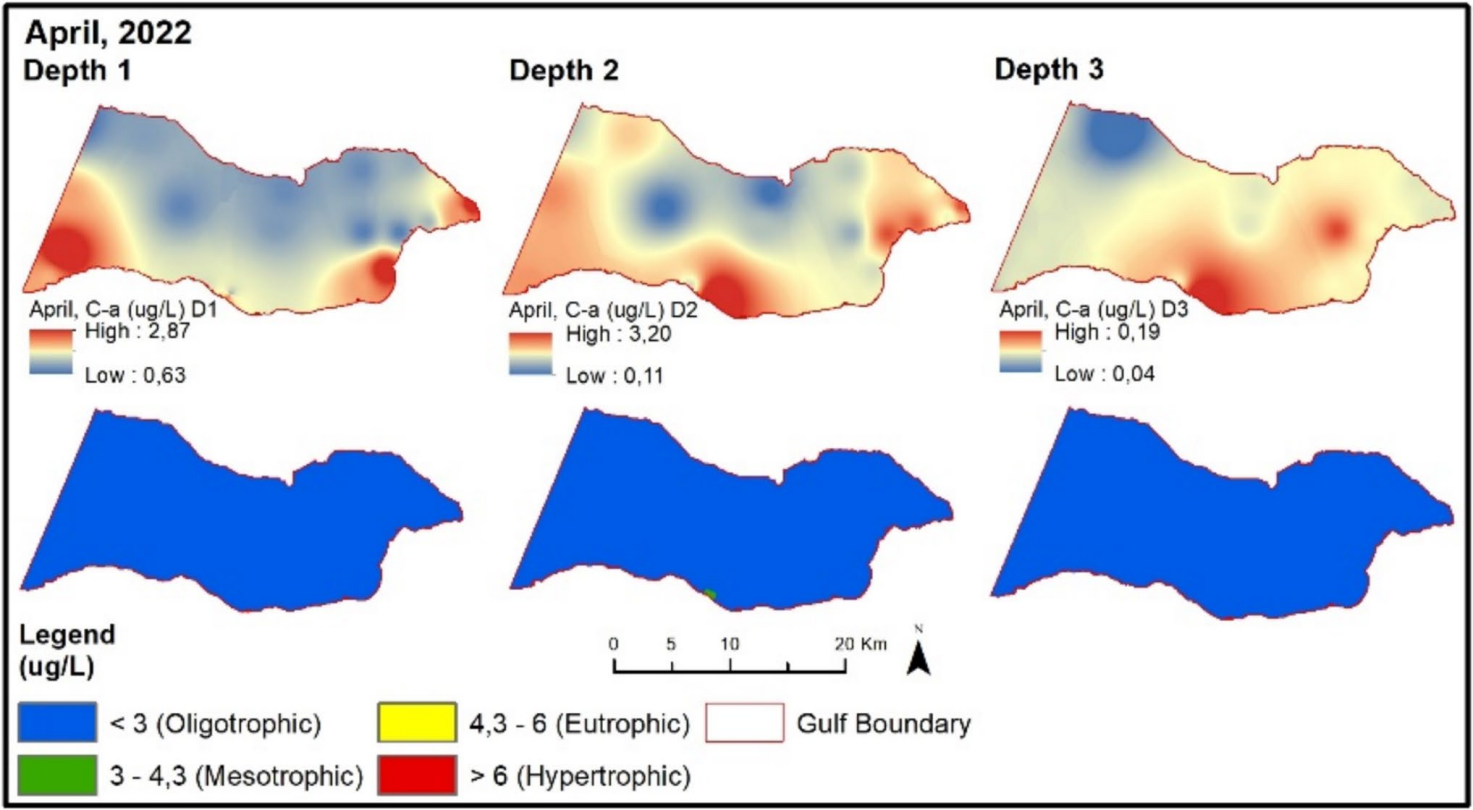
Evaluation of depth-dependent eutrophication criteria for the Gemlik Gulf in April 2022 (Depth 1: 0–5 m; Depth 2: 5–20 m; Depth 3: > 20 m)

In April 2022, NOx values are higher at points located south of the Gemlik Bay compared to points in the North (Fig. 8).

**Fig. 9:**
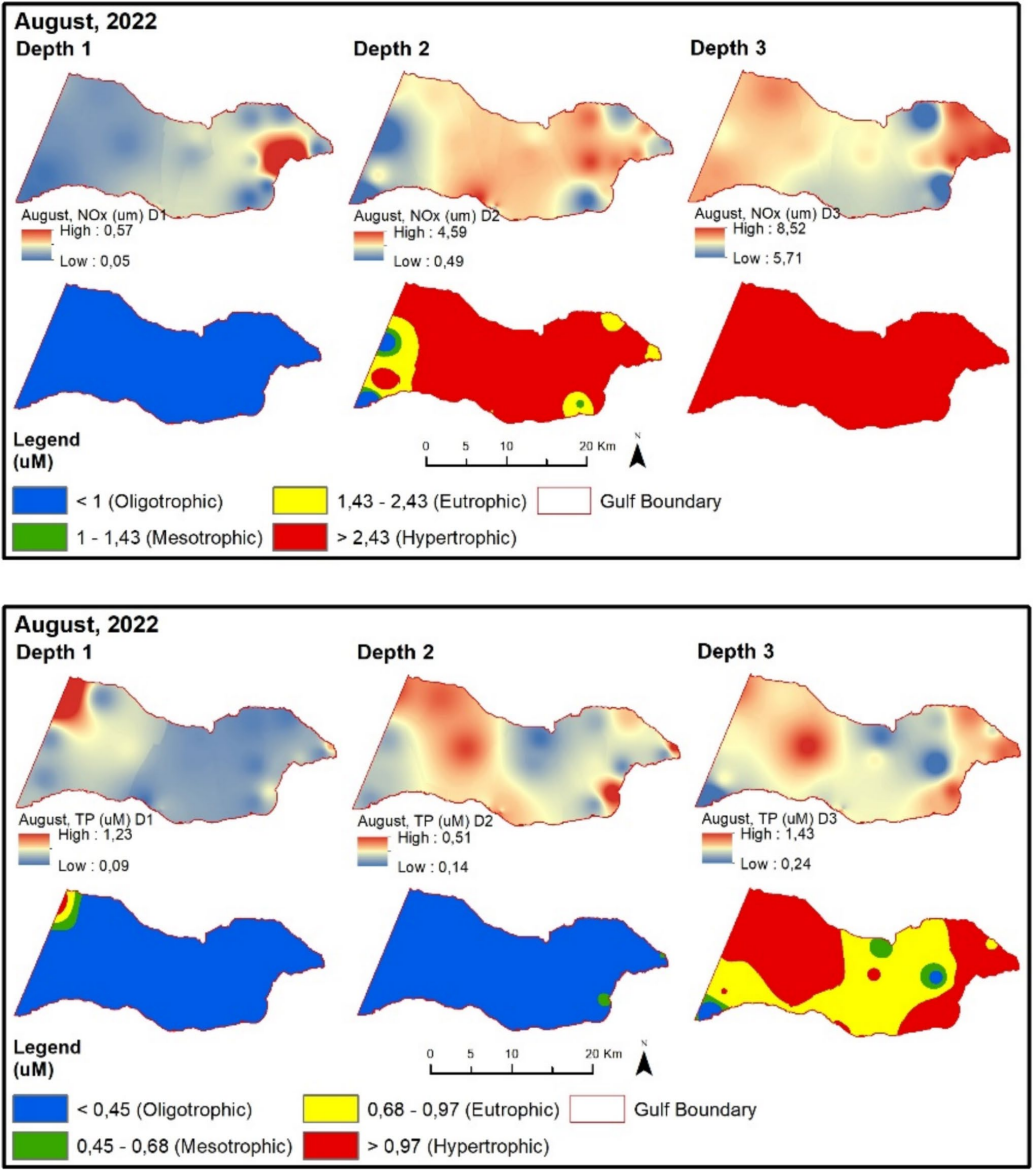

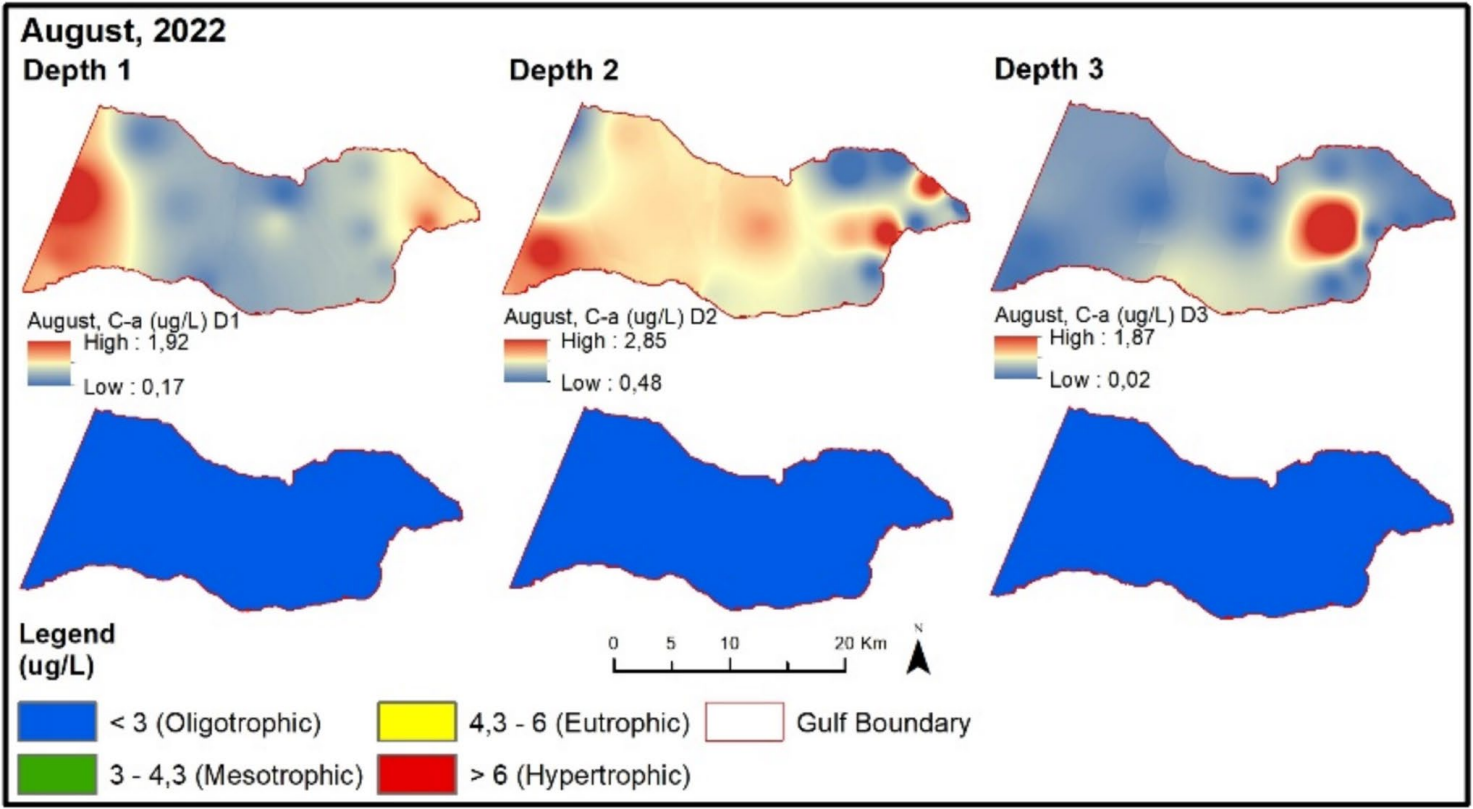
Evaluation of depth-dependent eutrophication criteria for the Gemlik Gulf in August 2022 (Depth 1: 0–5 m; Depth 2: 5–20 m; Depth 3: > 20 m)

TP values are higher at sampling points influenced by the Susurluk River and subjected to the pressure of urban wastewater in the inner part of the Gemlik Bay. The chlorophyll-a value exceeds the limit given in the regulation for the spring season compared to the winter season. Therefore, despite high chlorophyll-a values, almost all sampling points remain at oligotrophic levels according to the regulatory limit. In August 2022, NOx and TP values (except BBB9) are at oligotrophic levels (Fig. 9). Chlorophyll-a values are at low concentrations during this period. The secchi disk depth in the Gemlik Bay is 8.4 m on average during this period. In the bay, it mostly exceeds 6 m at oligotrophic levels. Points with lower depth show values below 6 m.

**Fig. 10:**
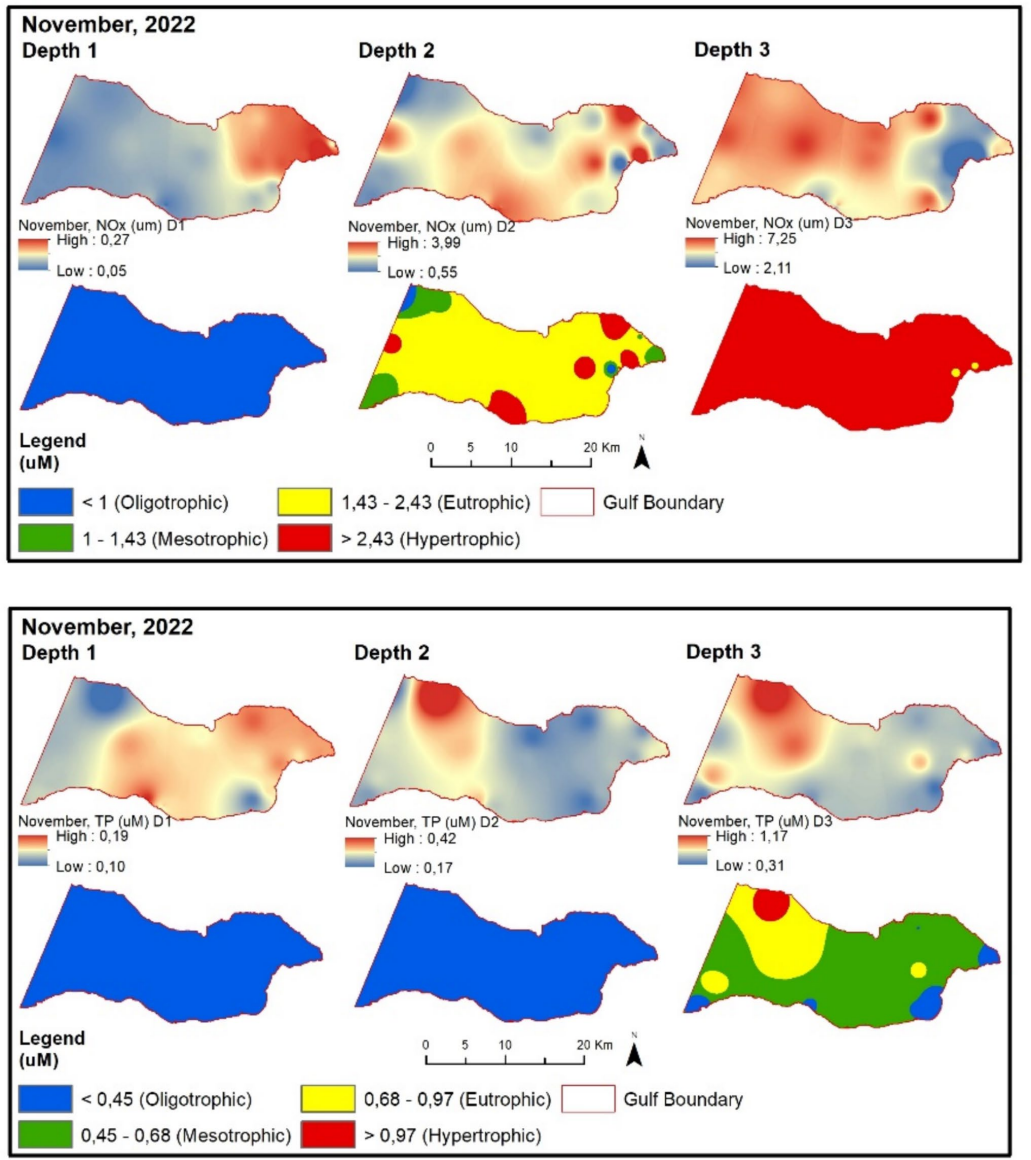

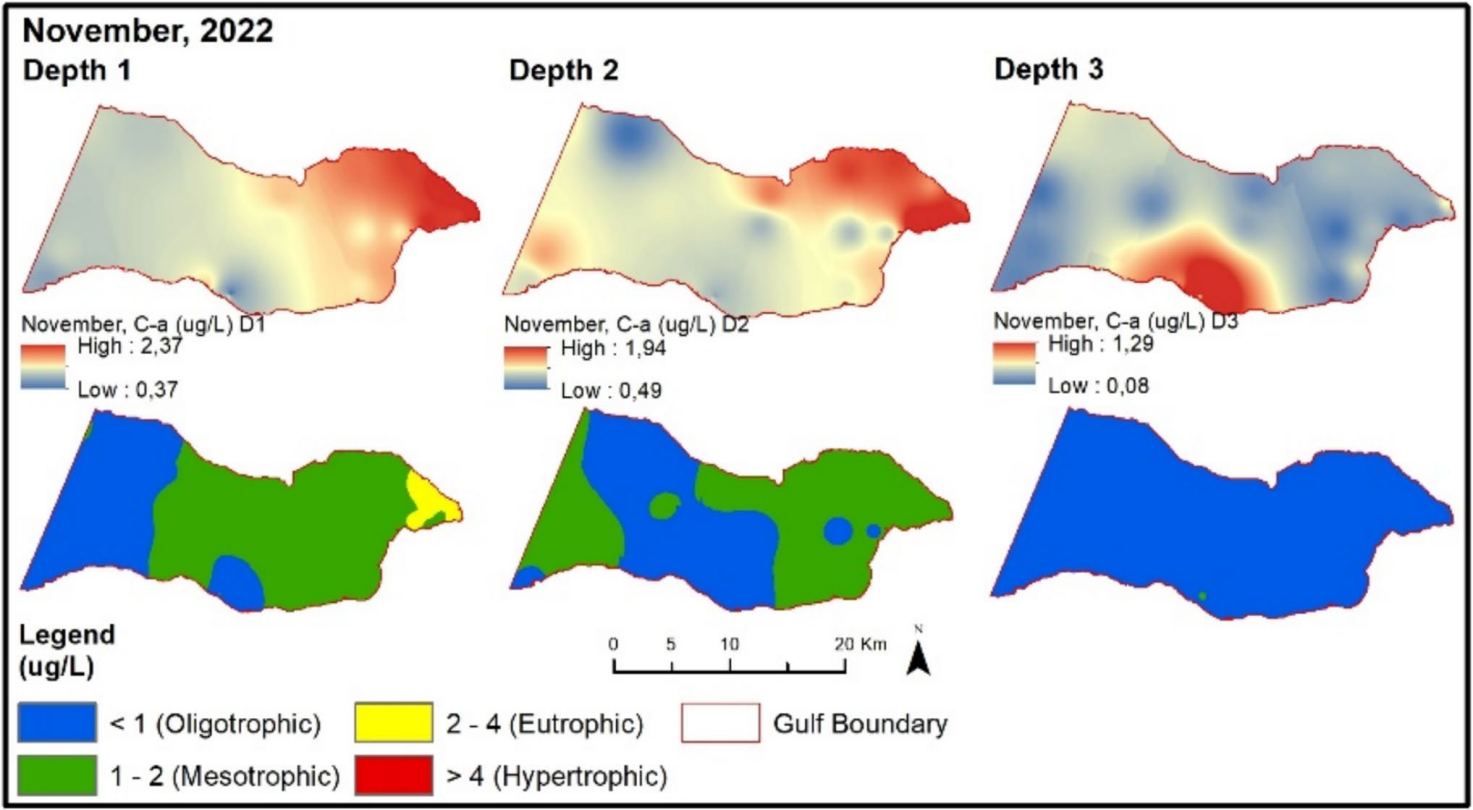
Evaluation of depth-dependent eutrophication criteria for the Gemlik Gulf in November 2022 (Depth 1: 0–5 m; Depth 2: 5–20 m; Depth 3: > 20 m)

In November 2022, NOx, TP, and secchi disk values are at oligotrophic levels. During this period, chlorophyll-a values are at a higher trophic level at points with settlements and urban wastewater treatment plants compared to other points (Fig. 10).

When evaluating the trophic status in relation to depth, it is evident that different eutrophication levels occur for each parameter. In terms of NOx concentrations, hypertrophic conditions tend to increase with depth, indicating an accumulation of nitrogenous compounds in deeper layers. For total phosphorus (TP), eutrophication appears to intensify in coastal areas, particularly during summer and autumn, suggesting a depth-related enrichment influenced by land-based nutrient inputs. In contrast, chlorophyll-a concentrations and trophic levels decrease with increasing depth, primarily due to reduced light penetration and limited phytoplankton activity in the lower water column. These findings highlight the parameter-specific and depth-dependent nature of eutrophication dynamics in the aquatic environment. The higher chlorophyll-a concentrations observed in surface waters can be primarily attributed to the availability of sunlight, which is essential for photosynthetic activity. As depth increases, light penetration decreases significantly, limiting photosynthesis and thereby reducing phytoplankton biomass and chlorophyll-a levels. Additionally, nutrient inputs from terrestrial sources, such as river discharges, wastewater, and surface runoff, tend to accumulate in surface layers, further enhancing primary productivity in the upper water column. In contrast, deeper layers generally experience limited light availability and lower phytoplankton growth, resulting in oligotrophic or mesotrophic conditions. This vertical distribution pattern reflects the combined effects of light attenuation, nutrient dynamics, and water column stratification.

Ignatiades (2005) stated that the ecological status of the environment is described by three trophic levels according to Chl a concentrations (oligotrophic, < 0.5 μg L^−1^; mesotrophic, 0.5–

1 μg L^−1^; eutrophic, > 1 μg L^−1^). In this context, the study area’s ecological quality status was likely eutrophic. In the Marmara Sea, phytoplankton, particularly diatoms, is the cause of the fall in nutrient levels during the spring and summer (Balkis and Balci 2010).

#### The pollution loads entering the Gulf

Engürücük Creek (EC), Karsak Creek (KC), Susurluk River (SR), and Nilüfer River (NR) flow into the Gemlik Gulf (Table S4). Engürücük Creek is a small stream that passes by the Kurşunlu Wastewater Treatment Plant. Its width is approximately 3 m, and the water level is generally less than 1 m. During some measurement periods, it has been observed that the creek is dry, with no flow.

Karsak Creek is a canalized river flowing through the Gemlik district and discharging into the Gemlik Bay. The width of the creek is around 17 m, and the water level is generally about 0.20 m. During periods of olive cultivation, an increase in salinity and conductivity values has been observed in Karsak Creek.

Susurluk River has the highest flow rate among the studied rivers. Due to the presence of agricultural fields around the river, it is an important watercourse for the region. The river discharges into the Sea of Marmara and has a width of 42–45 m with a water level of 6–8 m. The flow rate varies periodically.

Nilüfer River is a significant watercourse for the Bursa province, merging with the Susurluk River before flowing into the Sea of Marmara. During the sampling period, it was observed that the flow was slow. It is relatively more polluted compared to other rivers, with a width of around 20 m and a water level ranging between 1 and 2 m.

The pollutant loads carried by surface waters are presented in Table 3.

**Table 3:** Pollution loads transported by surface waters to the Gulf of Gemlik (BUSKİ 2022)

Parameters (kg day^−1^)	KC	EC	SR	NR
TN	3949.1	405.1	37,701.7	25,654.7
TP	698.5	16.2	6561.7	7737.3
NO_X_	112.3	9.74	4461.6	120.7
NH_4_-N	1117.1	5.58	12.6	10,425.9

Seasonal analyses indicated higher pollutant loads in September from Karsak Creek, in March from Engürücük Creek, between March and May for Susurluk River, and in winter months for Nilüfer River. In a general, it is observed that the Nilüfer and Susurluk River contribute a significant pollution load to the Gemlik Gulf (Tan 2021; Akdemir 2024; MoEU 2025). Rivers discharging into a sea have important roles in pollution, being affected from point and nonpoint sources, when considering factors such as the abundance of industrialization activity and high population density around it (Nausch et al. 1999). The data in the Table 3 show that the Susurluk River is the source carrying the highest overall pollutants, particularly in terms of total nitrogen and nitrate/nitrite. Nausch et al. (1999) reported that the amount of nutrients in surface waters increases with the high inflow of freshwater that carries large amounts of nitrogen in winter, when primary production is low. The Susurluk River carries the highest total nitrogen load to the Gemlik Gulf, while the Nilüfer River carries the highest total phosphorus load. While the Nilüfer River brings the highest total phosphorus load to the Gemlik Gulf, the Susurluk River also contributes a similarly high phosphorus load. In terms of nitrate and nitrite load, the Susurluk River makes the largest contribution. The highest contribution to the ammonium nitrogen load comes from the Nilüfer River. The Nilüfer River, on the other hand, is notable for its high ammonium nitrogen and total phosphorus loads. These two rivers carry the most pollution load to the Gemlik Gulf, while the Karsak and Engürücük Creeks contribute at lower levels.

Another source transporting pollutant load to the Marmara Sea is domestic wastewater treatment plants. When comparing the pollutant load carried by wastewater treatment plants and rivers in terms of nitrogen and phosphorus load, it is observed that the pollution originating from rivers is many times higher.

When the analyzed results of treated wastewater at discharge points of treatment plants are examined, pollution loads for Gemlik, Kurşunlu, Küçükkumla, and Mudanya treatment plants are determined as follows for TN: 56.55 kg day^−1^, 37.56 kg day^−1^, 50.29 kg day^−1^, and 65.55 kg day^−1^; for TP: 22.62 kg day^−1^, 5.008 kg day^−1^, 2.675 kg day^−1^, and 21.85 kg day^−1^, respectively. The discharge of treated wastewater from a depth of – 40 m is observed to dilute toward the surface, complying with the regulatory limit values. According to the analysis of samples taken from surface points where discharge into the sea occurs, the highest pollution load was observed during the winter months. Average deep-sea discharge analysis results for Gemlik, Küçükkumla Kurşunlu, and Mudanya locations are presented in Table 4.

**Table 4:** Analysis results of deep-sea discharge (BUSKİ 2022)

Parameters (mg day^−1^)	Gemlik	Küçük kumla	Mudanya	Kurşunlu
TN	3.99	0.95	4.27	0.05
TP	0.159	0.037	0.14	1.45
NO_X_	0.058	0.0096	0.17	0.02
NH_4_-N	0.151	0.019	0.16	0.04

It is observed that the TN and TP loads from domestic wastewater treatment plants are much lower compared to the pollution loads carried by rivers. This indicates that rivers carry significantly higher pollution to the Marmara Sea. According to the data, the wastewater treatment plant with the highest pollution load is the Mudanya treatment plant.

#### Water quality index

**Fig. 11:**
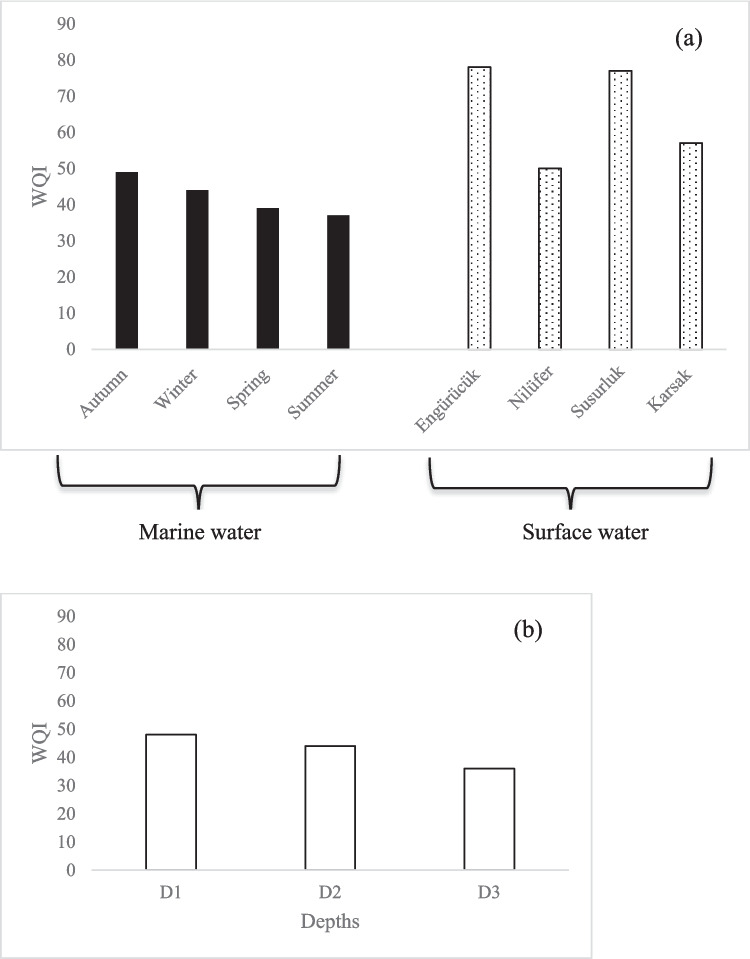
Water quality index (D1: depth of 0–5 m; D2: depth of 5–20 m; D3: depth of > 20 m)

Figure 11 depict the fluctuations in Water Quality Index (WQI) values and rates across all 31 stations within the investigated area. The calculated WQI values ranged between 37 and 49, reflecting medium to good water quality conditions. The period with the lowest water quality was during the summer seasons, with a general decline in water quality observed in the year 2022.

When the water quality index is calculated for surface waters (Fig. 11a), Engürücük Creek was determined to be 78 (very poor), Nilüfer River scored 50 (poor), Susurluk River scored 77 (very poor), and Karsak Creek scored 57 (poor). This situation indicates that the streams carry significant pollution to the Sea of Marmara.

As the depth of the sea (D1, D2, D3) increases, it is observed that the water quality improves (Fig. 11b). This finding indicates that the relatively lower water quality observed at the surface layer may be attributed to the influence of external pollutant inputs or anthropogenic pressures acting on the upper water column.

## Discussion

### Water temperature and pH

Water temperature is an important factor in determining water quality and analyzing aquatic habitats. It affects the distribution and living circumstances of aquatic species as well as a number of chemical and biological processes (Ustaoglu and Tepe 2019; Zaghloul et al. 2023).

Seasonal temperature analyses conducted in the Gemlik Gulf reveal that the thermal structure of the marine environment undergoes significant changes throughout the year. During winter and spring, vertical mixing is more pronounced across the water column, while cold water masses from the winter season persist at intermediate depths. Summer represents the period with the highest surface temperatures and the most distinct thermal stratification. During this time, vertical mixing is limited, and relatively stable temperature values prevail in the deeper layers. In autumn, as surface waters begin to cool, stratification starts to weaken, indicating a transitional phase influenced by external water masses. Seasonal temperature variations are shaped by vertical mixing, thermocline formation, freshwater inflows, and the influence of external water masses. These thermal dynamics not only govern the physical structure of the bay but also directly impact biological and chemical processes, playing a critical role in nutrient transport, oxygen distribution, and biological productivity.

In natural water systems, the pH level is another significant chemical parameter with biological significance. Certain compounds in water can be more or less dangerous depending on the pH (Boyd et al. 2016). The lowest pH values are usually seen in the winter, whereas the highest pH values are found in the summer. The summertime brings greater seawater pH levels because of more photosynthesis, warmer temperatures, less precipitation and runoff, and changes in ocean currents that promote mixing.

The exchange of carbon dioxide (CO_2_) with organic material in seawater is the cause of these seasonal fluctuations in environmental pH (Rudnick and Oviatt 1986; Oviatt 2004). Seawater pH rises throughout the winter and spring as a result of phytoplankton growth and a drop in net CO_2_ emissions; in the summer, however, it falls again as a result of higher CO_2_ levels in the sea (Hinga 1992; O’Boyle et al. 2012). As a result, variations in pH could be a sign of annual shifts in phytoplankton development. These elements cause the water’s carbon dioxide content to decrease, raising the pH levels as a result. The unprecedented scale and prolonged duration of the 2021 mucilage event accentuated the magnitude of its impact on marine ecosystems (Hekimoğlu and Gazioğlu 2021; Isinibilir et al. 2024). Marine mucilage is a mucus-like particulate organic substance found in the marine environment (Precali et al. 2005). It is primarily produced by phytoplankton, the initial component of biological production in the sea, which releases mucilage into the seawater when it proliferates under the influence of certain environmental factors. The Chl-a concentrations obtained in the study were also found to be high during the winter and spring seasons. Chl-a concentrations serves as an indicator of phytoplankton biomass, reflecting elevated productivity in these periods. During productive phases, increased CO₂ uptake by phytoplankton through photosynthesis can lead to a rise in pH levels (Mahaffey et al. 2020).

### Salinity

Salinity profiles show variations across the seasons, primarily due to seasonal changes and localized factors such as river input. Winter and summer exhibit more stable and consistent salinity profiles, while spring and autumn show greater variability, particularly in the intermediate layers, influenced by freshwater input and external water masses.

The unique properties of the Marmara Sea, including a stratified two-layer system that flows in reverse direction, are assumed to be responsible for fluctuations in temperature, salinity, and dissolved oxygen (DO) that were observed during the study period (Beşiktepe et al. 1994). The surface had the lowest salinity concentration in mid-summer. Stations with excessive salinity were identified as GDDD, KKDDD, and BBB 13, all of which are located in the inner gulf. Salinity distribution in this study was consistent, which can be attributed to vertical mixing in the water column during the autumn and winter seasons. Balcı and Balkıs (2017) observed a similar finding.

A substantial positive correlation (*p* < 0.01) was discovered between temperature and salinity throughout the water column, attributable to increased salinity with depth and the influence of Mediterranean-derived water. This is in line with the work of Aydogdu et al. (2023), who observed that salinity stratification in coastal areas is often linked to deeper, saltier water masses interacting with surface waters.

Indeed, Chiggiato et al. (2012) point to small-scale cyclonic eddies are widespread on the Marmara Sea's southern shelf and southeast regions, including the Gemlik Gulf. Divergence, or upwelling, is usually associated with these cyclonic eddies and can have a significant impact on the surrounding marine ecosystem. Their computer model also discovered an unusual upwelling of warm, briny water in the Gemlik Gulf.

Ustaoglu and Aydın (2020) also found an inverse association between dissolved oxygen (DO) and conductivity, indicating that DO levels fall as conductivity increases. Similar results were obtained in the present research.

### Dissolved oxygen

Dissolved oxygen levels consistently decrease with depth across all seasons, with notable declines observed in summer and autumn, reflecting the influence of external water masses and seasonal cooling. Surface oxygen levels remain higher during winter and spring, while more significant depletion is observed at depth during summer and autumn, particularly near river discharge areas.

Aquatic organisms rely on dissolved oxygen levels since low oxygen concentrations can produce hypoxia and anoxia in the water, resulting in fish deaths and other environmental issues (Zaghloul et al. 2023). Five ecological categories are assigned based on DO concentrations under the European Water Framework Directive (WFD): ≥ 5.7 mg L^−1^, classified as High; ≥ 4.0 < 5.7 mg L^−1^, classified as Good; ≥ 2.4 < 4.0 mg L^−1^, classified as Moderate; ≥ 1.6 < 2.4 mg L^−1^, classified as Poor; and < 1.6 mg L^−1^, classified as Bad (Best et al. 2007).

Based on dissolved oxygen (DO) levels, good-quality water status was only detected at depths of 30 and 50 m in this investigation, but high-quality water status was seen in the water column at depths ranging from 0.5 to 20 m. Aquatic ecosystem health is intimately correlated with nitrogen and phosphorus levels, which are essential for maintaining marine organisms (Abo-El-Khair et al. 2016).

### Nutrients

The levels of total nitrogen (TN) and total phosphorus (TP) are probably regulated by physical and biological processes. According to the investigation, TP and TN increases were correlated with salinity decreases, suggesting that sewage and industrial effluents contribute to coastal saltwater environments (Elsaye et al. 2019; Mahmoud et al. 2020).

At stations inside the gulf, surface nutrient concentrations rose noticeably due to freshwater inflow and enhanced Black Sea waters, especially during the early winter. These outcomes are most likely the result of numerous sources of treated and untreated sewage and industrial effluents being released into the environment.

The influx of freshwater with high nitrogen loading raises the concentrations of nutrients in surface waters during the winter months when primary production is low. Rivers that empty into the ocean are major sources of pollution, which is impacted by things like industrialization and dense population (Nausch et al. 1999).

Devlin and Brodie (2023) recently reported similar findings in another coastal system, where freshwater inputs significantly altered nutrient concentrations, leading to seasonal eutrophication.

Generally, the observed nutrient concentrations were controlled by combining seawater’s conservative and non-conservative characteristics (Manasrah et al. 2006). Salinity, water temperature, groundwater input, surface run off, atmospheric deposition, biological activities, and dissolved oxygen play an essential part in the different patterns of nutrients in coastal areas.

The Marmara sea water has become contaminated due to anthropogenic activities; as a result, organic matter from the land has precipitated from the surface to lower water levels, promoting microbial activity and the breakdown of organic matter (Cetecioğlu et al. 2009; Ritta et al. 2010). The presence of high nutrient levels, particularly phosphorus, suggests that these discharges are a significant contributor to eutrophication in the region. Suresh et al. (2023) recently highlighted the importance of managing wastewater discharges to mitigate nutrient loading and its ecological consequences in coastal waters.

One important ecological parameter influencing primary production and eutrophication is the nitrogen-to-phosphorus (N:P) ratio (I. Khedr et al. 2019). Water bodies with restricted N content have N:P molar ratios more than 5, while those with P deficiency have N:P molar ratios less than 5 (Okbah et al. 2017; Elsaye et al. 2019). Nitrogen limitation was evident in the Gemlik Gulf, where nitrogen contents were higher than phosphate concentrations, as shown by average N:P ratios ranging from 6.32 to 37.08.

According to earlier research (Aktan and Aykulu 2003; Tüfekçi et al. 2010), nitrogen is the Marmara Sea’s limiting nutrient. During the winter and early spring, N:P ratios can reach over 16. At MD19 station, the summer season had the highest N:P ratio (66.5), most likely as a result of early nutrient loading that promoted higher phytoplankton density (Cook et al. 2010). Conversely, phosphate concentrations were significantly low during the early summer period, contributing to the high N:P ratio rather than phosphate limitation.

In January, the highest N/P ratio was observed at stations GK2, BBB6, and MDDD, while in November, it was determined at stations GK2, MD19 A, and MD20. In April, the highest N/P ratio was determined at stations MD19 A, BBB7, and BB4, while in August, it was observed at stations KKDDD, MD22 A, and MD19.

This finding is consistent with the work of Vallina et al. (2023), who noted that seasonal shifts in nutrient availability and ratios can significantly impact phytoplankton dynamics and overall ecosystem productivity.

In general, nitrogen serves as the limiting factor for the Gemlik Gulf, whereas phosphorus acts as a significant contributor to eutrophication, primarily originating from domestic and industrial wastewater.

## Conclusion

This study highlights that coastal regions are transitional zones highly susceptible to human activities, which significantly impact marine ecosystems.

-The highest annual average concentrations of total phosphorus (TP) and total nitrogen (TN) were observed at nearshore stations and deep-sea discharge points (SD1, MD20, GDDD, KDDD).

-The Susurluk and Nilüfer Rivers are major contributors to nutrient inputs, significantly affecting the Gulf’s nutrient dynamics.

-While NOx levels in surface waters generally indicated oligotrophic conditions, hypertrophic trends were noted at greater depths due to nitrogen accumulation.

-TP concentrations increased markedly in coastal areas during summer and autumn, suggesting enhanced eutrophication influenced by land-based nutrient loads.

-Stations such as SD1, MD20, GDDD, and KKDDD were identified as high-risk zones, whereas GK1 and BBB12 were more stable and less impacted throughout the year.

Overall, the findings emphasize the urgent need for effective pollution control and sustainable monitoring strategies to preserve the ecological integrity of the Gemlik Gulf.

## Supplementary Information

Below is the link to the electronic supplementary material.

Supplementary Material 1 (DOCX 18.2 KB)

## Data Availability

The data that support the findings of this study are available from the corresponding author upon reasonable request.
