# Supplementary material for: Assessment of pollution status using Water Quality Index (WQI) and hydrochemical ındicators in the Gemlik Gulf, Marmara Sea, Türkiye: a spatial and temporal perspective
Source: Environ Sci Pollut Res Int. 2025 Jun 2;32(24):14860–90. doi: 10.1007/s11356-025-36560-8 (PMC12202698; doi:10.1007/s11356-025-36560-8)
Supplement: Supplementary file 1 — Supplementary Material 1 (DOCX 18.2 KB) [file 11356_2025_36560_MOESM1_ESM.docx]

**Table S1.** Sampling Points (SP) for Marine Water Quality Monitoring Study

| **No of SP** | **Code of SP** | **Coordinates**  **N E** | | **No of SP** | **Code of SP** | **Coordinates**  **N E** | |
| --- | --- | --- | --- | --- | --- | --- | --- |
| 1 | MD89A-GDDD | 40° 25.657' | 29° 08.028' | 17 | BBB1 | 40° 26.202' | 29° 08.855' |
| 2 | KUMDE-KKDDD | 40° 28.265' | 29° 05.295' | 18 | BBB2 | 40° 27.165' | 29° 07.150' |
| 3 | MD88 | 40° 27.904' | 29° 02.421' | 19 | BBB3 | 40° 25.469' | 29° 06.259' |
| 4 | MD22A | 40° 22.920' | 28° 53.070' | 20 | BBB4 | 40° 25.041' | 29° 04.581' |
| 5 | MD22 | 40° 26.422' | 28° 51.651' | 21 | BBB5 | 40° 25.101' | 29° 02.452' |
| 6 | MDSD | 40° 24.494' | 28° 44.628' | 22 | BBB6 | 40° 25.488' | 28° 57.251' |
| 7 | MDSD1 | 40° 23.280' | 28° 43.650' | 23 | BBB7 | 40° 26.962' | 28° 57.814' |
| 8 | GK1 | 40° 27.070' | 28° 45.080' | 24 | BBB8 | 40° 22.435' | 28° 54.489' |
| 9 | GK2 | 40° 29.704' | 28° 49.648' | 25 | BBB9 | 40° 30.336' | 28° 45.507' |
| 10 | MD19A | 40° 32.195' | 28° 25.462' | 26 | BBB10 | 40° 30.921' | 28° 42.103' |
| 11 | MD20 | 40° 23.134' | 28° 35.008' | 27 | BBB11 | 40° 22.245' | 28° 38.012' |
| 12 | SD2 | 40° 24.480' | 28° 31.324' | 28 | BBB12 | 40° 25.497' | 28° 20.746' |
| 13 | SD1 | 40° 24.116' | 28° 31.063' | 29 | BBB13 | 40° 31.336' | 28° 38.368' |
| 14 | MD19 | 40° 24.619' | 28° 26.929' | 30 | KDDD | 40° 23.382' | 29° 03.685' |
| 15 | SD3 | 40° 26.830' | 28° 33.574' | 31 | MDDD | 40° 22.298' | 28° 54.375' |
| 16 | MD90 | 40° 22.654' | 29° 02.141' |  |  |  |  |

**Table S2. Relative weights and normalization factors of 7 parameters.**

| Variable | Relative weights | Normalization factor (C_i_) | | | | | | | | | | |
| --- | --- | --- | --- | --- | --- | --- | --- | --- | --- | --- | --- | --- |
|  | | 100 | 90 | 80 | 70 | 60 | 50 | 40 | 30 | 20 | 10 | 0 |
| T | 1 | 21/16 | 22/15 | 24/14 | 26/12 | 28/10 | 30/5 | 32/0 | 36/-2 | 40/-4 | 45/-6 | >45/<-6 |
| pH | 1 | 7 | 7-8 | 7-8.5 | 7-9 | 6.5-7 | 6-9.5 | 5-10 | 4-11 | 3-12 | 2-13 | 1-14 |
| DO | 4 | ≥7.5 | >7 | >6.5 | >6 | >5 | >4 | >3.5 | >3 | >2 | ≥1 | <1 |
| TP | 1 | <0.2 | <1.6 | <3.2 | <6.4 | <9.6 | <16 | <32 | <64 | <96 | ≤160 | >160 |
| NO_2_ | 2 | <0.005 | <0.01 | <0.03 | <0.05 | <0.1 | <0.15 | <0.2 | <0.25 | <0.5 | ≤1 | >1 |
| NO_3_ | 2 | <0.5 | <2 | <4 | <6 | <8 | <10 | <15 | <20 | <50 | ≤100 | >100 |
| NH_4_-N | 3 | <0.01 | <0.05 | <0.1 | <0.2 | <0.3 | <0.4 | <0.5 | <0.75 | <1 | ≤1.25 | >1.25 |

Parameters from Pesce and Wunderlin (2000), Kannel et al. (2007) and Koçer and Sevgili (2014).

**Table S3.** Eutrophication Criteria for Coastal Waters of the Marmara Sea (Surface Water Quality Management Regulation, SWQMR 2016)

| **Water Quality Class** | **TP** | | **NO_x_** | | **Chlorophyll-a (µg/L) (Spring)** | **Chlorophyll-a (µg/L) (Autumn)** | **Secchi Disk Depth (m)** |
| --- | --- | --- | --- | --- | --- | --- | --- |
|  | **µg/L** | **µM** | **µg/L** | **µM** |  |  |  |
| Oligotrophic | <14 | <0.45* | <14 | 1* | <3 | <1 | >6 |
| Mesotrophic | 21 | 0.68* | 20 | 1.43* | 4.3 | 2 | 4.5 |
| Eutrophic | 30 | 0.97* | 34 | 2.43* | 6 | 4 | 3 |
| Hypertrophic | >30 | >0.97* | >34 | >2.43* | >6 | >4 | <3 |

*The µg/L values specified in the regulation have been converted to µM. µM: (µmol/L)

NOx: Oxidized nitrogen compounds (NO₃-N + NO₂-N).

**Table S4. The coordinates of the stream that flow into the Gulf of Gemlik.**

| **Name of Stream** | **Coordinates**  **N E** | |
| --- | --- | --- |
| Karsak Creek | 40° 25.442' K | 29° 09.478' D |
| Engürücük Creek | 40° 23.482' K | 29° 04.278' D |
| Susurluk River | 40° 17.318' K | 28° 26.015' D |
| Nilüfer River | 40° 17.771' K | 28° 27.520' D |
